# Chamber-specific regulation of myofilament Ca^2+^ sensitivity by nNOSβ via troponin I phosphorylation: Predominance in the right ventricle

**DOI:** 10.1085/jgp.202613982

**Published:** 2026-10-05

**Authors:** Jae Won Kwon, Hyerim Park, Ryeon Heo, Jihyun Jang, Jintae Kim, Seung Beom Oh, Yin Hua Zhang, Sung Joon Kim, Young Keul Jeon

**Affiliations:** 1 Department of Physiology, Seoul National University College of Medicine, Seoul, Republic of Korea; 2 Department of Biomedical Sciences, Seoul National University College of Medicine, Seoul, Republic of Korea; 3 Ischemic/Hypoxic Disease Institute, Seoul National University College of Medicine, Seoul, Republic of Korea; 4 Center for Cardiovascular Research, Abigail Wexner Research Institute, Nationwide Children’s Hospital, Columbus, OH, USA; 5 Department of Biochemistry & Molecular Biology, Hanyang University College of Medicine, Seoul, Republic of Korea

## Abstract

Neuronal nitric oxide (NO) synthase (nNOS) is an important regulator of excitation–contraction coupling in cardiomyocytes, yet its chamber-specific role in right ventricular cardiomyocytes (RVCMs) remains incompletely understood. In this study, we compared the effects of nNOS inhibition on contractile function, Ca^2+^ signaling, and myofilament-associated regulation in RVCMs and left ventricular cardiomyocytes (LVCMs). Pharmacological inhibition of nNOS with S-methyl-L-thiocitrulline (SMTC) enhanced sarcomere shortening and impaired relaxation in both cell types, with markedly greater effects observed in RVCMs. In contrast, SMTC-induced changes in Ca^2+^ transients and L-type Ca^2+^ current were more pronounced in LVCMs than in RVCMs, indicating a dissociation between Ca^2+^ signaling and mechanical responses in the SMTC-treated RVCMs. Analysis of dynamic Ca^2+^-sarcomere length coupling indicated that nNOS inhibition in RVCMs preferentially altered the relationship between intracellular Ca^2+^ signaling and sarcomere response, particularly during relaxation, rather than simply reflecting changes in Ca^2+^ magnitude. Consistent with this interpretation, inhibition of nNOS reduced cardiac troponin I (cTnI) Ser23/24 phosphorylation and was associated with altered endogenous Ca^2+^ buffering properties in RVCMs. Moreover, myofilament-enriched fractions from RVCMs exhibited higher levels of Ser1417-phosphorylated nNOSβ. Collectively, these data indicate that myofilament-associated nNOSβ predominantly regulates RV contractility through cTnI phosphorylation-mediated Ca^2+^ desensitization.

## Teaser

This study shows that myofilament-associated nNOS preferentially restrains right ventricular contraction by regulating Ca^2+^-binding affinity through troponin I phosphorylation, revealing a chamber-specific mechanism that modulates calcium signaling and mechanical output in the heart.

## Introduction

Nitric oxide (NO) is a well-established signaling molecule with a wide range of physiological effects, including a critical role in regulating cardiovascular function. Among the three NO synthase (NOS) isoforms (neuronal NOS [nNOS or NOS1], cytokine-inducible NOS [NOS2], and endothelial NOS [eNOS or NOS3]), nNOS plays a pivotal role in excitation–contraction (E-C) coupling and calcium handling in cardiomyocytes (Shah and MacCarthy, 2000; Sears et al., 2003).

In cardiomyocytes, nNOS is spatially localized near the SR and the plasma membrane. This compartmentalization enables precise modulation of calcium dynamics via posttranslational modification of key enzymes involved in Ca^2+^ release and uptake (Xu et al., 1999; Carnicer et al., 2017). Specifically, nNOS-derived NO modulates ryanodine receptor (RyR2) function; while it can enhance SR Ca^2+^ leak under pathological conditions, it also provides essential physiological regulation for normal Ca^2+^ signaling and contraction-relaxation coupling (Gonzalez et al., 2007; Vielma et al., 2016). Furthermore, nNOS influences cardiomyocyte contractility and relaxation (lusitropy) by affecting L-type calcium channel (LTCCs) current (*I*_Ca,L_) and phospholamban activity (Zhang et al., 2014).

Mechanistically, nNOS-derived NO stimulates cGMP production via stimulating guanylate cyclase, leading to the cGMP-dependent PKG pathways, promoting phosphorylation of phospholamban and subsequent enhancement of SR calcium reuptake via SR/ER Ca^2+^-ATPase (SERCA) 2a (Massion et al., 2003; Carnicer et al., 2013; Zhang et al., 2014). Additionally, NO can directly modify signaling molecules via S-nitrosylation of cysteine residues, altering protein functions responsible for cardiac E-C coupling and contractility (Lima et al., 2010).

Previous studies using both genetic deletion of nNOS (nNOS^−/−^) and pharmacological inhibition have demonstrated that loss of nNOS activity leads to prolonged relaxation and a slower rate of cytosolic Ca^2+^ decay due to reduced phospholamban phosphorylation, suggesting that nNOS is a key regulator of cardiac lusitropy (Massion et al., 2003; Carnicer et al., 2013). In addition, nNOS inhibition has been reported to increase the amplitude of *I*_Ca,L_, indicating a negative inotropic effect under physiological conditions (Sears et al., 2003). Importantly, nNOS is also associated with myofilaments, where it exerts compartmentalized signaling to facilitate myofilament Ca^2+^ desensitization and relaxation, a mechanism notably observed in hypertensive rats (Kojda and Kottenberg, 1999; Massion et al., 2003; Sears et al., 2003; Zhang et al., 2014).

Despite these extensive studies, our understanding of nNOS in cardiac physiology and pathophysiology has been largely limited to left ventricular cardiomyocytes (LVCMs), with comparable investigations in right ventricular cardiomyocytes (RVCMs) notably lacking. The right ventricle (RV), characterized by a thinner wall and crescent shape, is adapted to pump blood into the low-resistance pulmonary circulation. Although the RV and left ventricle (LV) eject the same stroke volume, they operate under markedly different mechanical conditions. The normal RV exhibits a triangular pressure–volume (P–V) relationship, with ejection beginning during pressure development and continuing during pressure decline, whereas the LV has more clearly defined isovolumic phases in the P–V relation (P–V loop) of the cardiac cycle (Redington et al., 1988). In addition, RV free-wall contraction has a greater longitudinal or meridional component, in contrast to the more prominent circumferential shortening of the LV lateral wall (Naito et al., 1995). Thus, cardiomyocytes in the two chambers experience distinct temporal and directional patterns of mechanical loading. Recent findings in isolated cardiomyocytes, including our own, suggest that normal RVCMs differ significantly from LVCMs in several aspects, such as action potential duration, E-C coupling efficiency, troponin expression patterns, and intracellular calcium buffering capacity (Kondo et al., 2006; Molina et al., 2016; Jeon et al., 2022).

In cardiac myocytes, intracellular Ca^2+^ mediates E-C coupling by binding to cardiac troponin (cTn) C (cTnC), the Ca^2+^-sensing subunit of the cTn complex. The cTn complex also includes cTnI, which inhibits actin–myosin interaction, and cTnT, which anchors the complex to tropomyosin. Ca^2+^-binding to cTnC relieves cTnI-mediated inhibition, permitting crossbridge formation and contraction. Beyond initiating contraction, cTnC functions as a major cytosolic Ca^2+^ buffer. This myofilament Ca^2+^ buffering critically influences the magnitude and decay kinetics of Ca^2+^ transients, thereby shaping contractile performance. In our previous work, we demonstrated that RVCMs exhibit a lower myofilament Ca^2+^ buffering capacity than LVCMs, primarily due to reduced expression of the cTn complex (Jeon et al., 2022). However, how this buffering capacity is modulated by physiological stimuli remains unclear.

The affinity of cTnC for Ca^2+^ is modulated by posttranslational modifications of cTnI; specifically, cTnI phosphorylation (p-cTnI) decreases myofilament Ca^2+^ sensitivity and facilitates Ca^2+^ dissociation from cTnC (Kooij et al., 2010; Salhi et al., 2014). Myofilament-localized nNOS has been implicated in regulating p-cTnI, positioning it as a key modulator of myofilament Ca^2+^ sensitivity (Kaye et al., 1999; Zhang et al., 2014). While the role of nNOS in myofilament sensitivity is recognized, the quantitative relationship between sensitivity and buffering capacity, which are related but distinct properties, has not been directly established (Eisner et al., 2017). Moreover, the chamber-specific effects of nNOS on functional myofilament Ca^2+^ sensitivity and endogenous Ca^2+^ buffering, potentially through regulation of the troponin complex, remain unexplored. Although we previously identified basal differences in Ca^2+^ buffering between RVCMs and LVCMs (Jeon et al., 2022), the specific regulation of this buffering by nNOS signaling in the RV versus the LV has yet to be elucidated.

In the present study, we investigated the role of nNOS in freshly isolated cardiomyocytes by combining pharmacological inhibition using S-methyl-L-thiocitrulline (SMTC) with simultaneous analysis of sarcomere length (SL) dynamics and E-C coupling. We also compared *I*_Ca,L_ via whole-cell patch-clamp recordings and assessed protein phosphorylation by immunoblot analysis. Intriguingly, SMTC treatment revealed a distinct phenotype in RVCMs compared with LVCMs: nNOS inhibition led to a more pronounced increase in contractility in RVCMs, despite a blunted increase in *I*_Ca,L_ and intracellular Ca^2+^ transients. This discrepancy suggests that nNOS inhibition primarily affects myofilament sensitivity rather than Ca^2+^ influx in the RVCMs. Consistent with this hypothesis, we observed a significant nNOS-dependent modulation of Ca^2+^ sensitivity and buffering capacity specifically in RVCMs. Furthermore, RVCMs exhibited higher levels of myofilament-associated nNOSβ and a more prominent nNOS-dependent regulation of p-cTnI. The physiological implications of these chamber-specific mechanisms are discussed.

## Materials and methods

### Isolation of ventricular cardiomyocytes

Ventricular cardiomyocytes were isolated from male Sprague–Dawley rats (RRID:RGD_1566457). All experimental procedures were conducted in accordance with the Guide for the Care and Use of Laboratory Animals published by the US National Institutes of Health (NIH Publication No.: 85-23, revised 1996) and were approved by the Institutional Animal Care and Use Committee of Seoul National University (IACUC approval No.: SNU-160128-1). A total of 55 rats were used. At the time of experimentation, rats were 12 wk old with a mean body weight of 322.7 ± 8.95 g.

Cardiomyocytes were isolated using a standard enzymatic dissociation protocol as previously described (Jin et al., 2012), with minor modifications. Rats were anesthetized by intraperitoneal injection of ketamine (90 mg·kg^−1^) and xylazine (10 mg·kg^−1^). Adequate depth of anesthesia was confirmed by absence of a toe-pinch reflex. Hearts were rapidly excised and mounted on a Langendorff perfusion system maintained at 37°C. Hearts were perfused at a constant flow rate (8–10 ml·min^−1^) according to the following sequence: (1) 8 min with Ca^2+^-free Tyrode solution; (2) 8 min with Ca^2+^-free Tyrode solution containing collagenase (1 mg·ml^−1^; Worthington Biochemical Co.), protease (0.133 mg·ml^−1^), BSA (1.65 mg·ml^−1^), and Ca^2+^ (0.05 mM); and (3) an additional 10 min digestion with collagenase-containing Tyrode solution. The interventricular septum was excluded from analysis. To ensure the homogeneity of the RVCMs and LVCMs, only tissue samples from the free walls of the RVs and LVs were collected, excluding the interventricular septum. Isolated cardiomyocytes were resuspended either in cardioplegic storage solution for contractility measurements or in Kraft–Brühe (K-B) solution for electrophysiological experiments (Table 1). The yield of rod-shaped, calcium-tolerant cardiomyocytes ranged from 60% to 80%. Cardiomyocytes designated for physiological experiments were used immediately after isolation, whereas those intended for biochemical analyses were centrifuged and stored at −80°C until further use. For SMTC treatment, cardiomyocytes were preincubated with 100 nM SMTC for 20 min prior to the start of experiments, and the same concentration of SMTC was maintained in all perfusion solutions throughout data acquisition.

**Table 1.** Solutions used in the present study (in mM)

​	K-B	NT	Cs-pipette	*I*_Ca,L_-pipette	0Na/0Ca	0Na/2Ca
NaCl	118	141.4	​	​	​	​
LiCl	​	​	​	​	141.4	141.4
CsCl	​	​	140.0	140.0	​	​
KCl	4.7	4.0	​	​	4.0	4.0
HEPES	​	10.0	10.0	10.0	10.0	10.0
NaHCO_3_	25.0	​	​	​	​	​
EGTA	​	​	​	0.1	0.1	0.1
NaH_2_PO_4_	​	0.33	​	​	​	​
KH_2_PO_4_	1.2	​	​	​	​	​
MgSO_4_	1.1	​	​	​	​	​
MgATP	​	​	4.0	​	​	​
MgCl_2_	​	1.0	1.0	​	5.0	1.0
CaCl_2_	1.8	1.8	​	​	​	2.0
Glucose	5.6	5.5	​	​	5.5	5.5
Mannitol	10.0	14.5	​	​	​	​
pH	7.4	7.4	7.2	7.2	7.4	7.4

### NO measurement via Griess assay

NO production was indirectly assessed by quantifying nitrite levels in cardiomyocytes using a colorimetric NO assay kit based on the Griess Reagent System (Promega). After homogenization, lysates were centrifuged to collect the supernatant, and total protein concentration was determined using the Bradford assay (Bio-Rad). For nitrite measurement, 50 μl of each supernatant was transferred to a 96-well microplate and mixed with 50 μl of sulfanilamide solution, followed by incubation for 5–10 min at room temperature in the dark. Subsequently, 50 μl of N-(1-naphthyl)ethylenediamine dihydrochloride (Sigma-Aldrich) was added to each well, and the reaction was allowed to proceed under the same conditions. The resulting azo dye, proportional to nitrite concentration, was quantified by measuring absorbance at 540 nm using a microplate spectrophotometer (PowerWave XS; BioTek Instruments). A standard curve was generated using serial dilutions of sodium nitrite (Sigma-Aldrich), and sample nitrite concentrations were calculated by interpolation from this curve.

### Isolation of membrane/cytosolic and myofilament-enriched fraction

Fractionation of cardiomyocytes for immunoblotting was performed using two distinct protocols, as previously described (Jang et al., 2015). This fractionation procedure was previously shown to yield a myofilament-enriched fraction with no detectable GAPDH contamination, as demonstrated in Fig. 5 A of Jang et al. (2015). To obtain membrane and cytosolic fractions, cells were lysed in buffer containing 25 mM Tris-HCl (pH 7.4), 150 mM NaCl, 0.5 mM EGTA, and 1% Triton X-100 supplemented with protease and phosphatase inhibitor cocktails (Roche). The lysates were centrifuged at 15,000 rpm for 30 min, and the supernatant containing soluble membrane and cytosolic proteins was collected for further analysis. For isolation of the myofilament fraction, cardiomyocytes were rinsed with ice-cold phosphate-buffered saline and transferred to screw-cap microtubes containing 2.5-mm zirconia/silica beads and cold extraction buffer (60 mM KCl, 30 mM imidazole, 2 mM MgCl_2_, and 1% Triton X-100, supplemented with protease and phosphatase inhibitors). Cells were homogenized using a Mini-Beadbeater-8 (BioSpec Products) for 20 s, followed by centrifugation at 8,000 rpm for 10 min at 4°C. The supernatant was discarded, and the homogenization-centrifugation cycle was repeated at least six times to ensure efficient removal of soluble proteins. The remaining pellet, enriched in myofilament proteins, was resuspended in high-salt buffer (Bio-Rad) and used for subsequent biochemical analyses.

### Immunoblotting

Protein concentrations were determined using the Bradford assay (Bio-Rad). Equal amounts of protein were denatured at 95°C for 5 min in sample buffer, separated by SDS-PAGE, and transferred to polyvinylidene difluoride membranes (Bio-Rad) using transfer buffer containing 25 mM Tris, 192 mM glycine, 0.01% SDS, and 20% methanol. Membranes were blocked for 1 h at room temperature in TBS with 0.1% Tween-20 containing 5% BSA. Membranes were incubated overnight at 4°C with primary antibodies against nNOS (RRID:AB_304965, Abcam), phospho-nNOS (RRID:AB_304964, Abcam), eNOS (RRID:AB_397690, BD Biosciences), phospho-eNOS^Thr495^ (RRID:AB_2153176, Cell Signaling Technology), phospho-eNOS^Ser1177^ (RRID:AB_823493, Cell Signaling Technology), TnC (RRID:AB_306435, Abcam), TnI (RRID:AB_2206278, Cell Signaling), phospho-TnI (RRID:AB_2206275, Cell Signaling Technology), TnT (RRID:AB_261723, Sigma-Aldrich), and α-actin (RRID:AB_476695, Sigma-Aldrich). After washing, membranes were incubated with appropriate HRP-conjugated secondary antibodies. Immunoreactive bands were detected using enhanced chemiluminescence (ECL Plus; Merck) and quantified by densitometry. Protein levels were normalized to α-actin. Each sample represented an independent biological replicate from a different animal. Samples were excluded if α-actin levels varied by >10% between left and right ventricular lysates from the same animal. The phosphorylation site is referred to as Ser^1417^ according to the antibody nomenclature; the homologous site is commonly designated Ser^1412^ in rodent nNOS literature.

### SL and [Ca^2+^]_c_ measurements using IonOptix system

The physiological properties of isolated cardiomyocytes were assessed using the IonOptix system (IonOptix). Contractile function was evaluated by measuring SL shortening in response to electrical field stimulation (EFS) at a frequency of 2 Hz. The SL was calculated in real time using the SarcLen module, which analyzes periodic striation patterns within the cell via fast Fourier transform of the optical density profile across the cell. Freshly isolated cardiomyocytes were transferred into a bath mounted on the stage of an inverted microscope (Diaphot 300; Nikon). The bath (0.15 ml) was continuously perfused with normal Tyrode solution (NT, Table 1) at a constant rate (5 ml·min^−1^). All experiments were performed at 37 ± 1°C. For ratiometric fluorescence measurements using fura-2, cardiomyocytes were incubated with 2 μM fura-2 acetoxymethyl ester for 15 min at room temperature. Following gravity sedimentation, the supernatant was carefully removed, and the cells were resuspended in a perfusion buffer containing 500 μM Ca^2+^ for 10 min to allow for intracellular de-esterification of the dye. The fura-2–loaded cardiomyocytes were then transferred to a temperature-controlled imaging chamber (37 ± 1°C) and alternately illuminated with excitation wavelengths of 360 and 380 nm using a dual-wavelength excitation system. The corresponding emission signals were detected in real time using photomultiplier tubes (PTIs). The ratio of fluorescence intensities (F_360_/F_380_) was continuously monitored as a surrogate index of [Ca^2+^]_c_ dynamics. For each condition, F_360_/F_380_ was averaged over a minimum of 20 steady-state contraction cycles per cardiomyocyte.

### Electrophysiology

Freshly isolated cardiomyocytes were transferred to a recording chamber mounted on the stage of an inverted microscope (Ti, Nikon). The chamber (volume: 0.15 ml) was continuously perfused at a constant rate of 5 ml·min^−1^, and voltage-clamp experiments were performed at room temperature (22–25°C). Conventional and Nystatin-perforated whole-cell patch-clamp recordings were obtained using borosilicate glass pipettes with a tip resistance of ∼2.5 MΩ. Series resistance, estimated from the time constants of the capacitive transients, was kept below 10 MΩ in the whole-cell configuration (calcium imaging) and 16 MΩ in the perforated whole-cell configuration. LTCC current was recorded using the nystatin-perforated whole-cell patch-clamp technique with an *I*_Ca,L_ pipette solution (Table 1). To normalize for cell size, current amplitudes were divided by cell capacitance and expressed as pA·pF^−1^. Patch pipettes were connected to the CV-203BU headstage of an Axopatch 200B patch-clamp amplifier (Molecular Devices). Data acquisition and command pulse generation were performed using pCLAMP software (version 10.6.2) in conjunction with a Digidata 1440A interface (Molecular Devices). Currents were sampled at 10 kHz and low-pass filtered at 5 kHz using a Bessel filter. Offline data analysis was performed using Clampfit software (version 11.0.1; Molecular Devices).

### Calcium imaging technique for measuring Ca^2+^-binding ratio

As in our previous research (Jeon et al., 2022), Ca^2+^ buffering capacity was assessed using simultaneous whole-cell patch-clamp recording and fura-2 ratiometric fluorescence imaging. Briefly, cardiomyocytes were dialyzed with a Cs^+^-based pipette solution (Cs-pipette; Table 1) supplemented with 50 µM fura-2 pentapotassium salt, and fluorescence signals were monitored using alternating 340/380 nm excitation with emission collected at 510 nm via a PTI.

To estimate endogenous cytosolic Ca^2+^ buffering, we defined the intrinsic Ca^2+^-binding ratio ($\kappa_{S}$) in terms of a hypothetical endogenous Ca^2+^-binding species “S” (Mathias et al., 1990). The total Ca^2+^-binding ratio including fura-2 ($\kappa_{B}$) and the endogenous binding ratio ($\kappa_{S}$) were defined as follows:

[CaB]=[CaS]+[Cafura2],

$$\kappa_{S}\equiv \frac{d\left[CaS\right]}{d\left[Ca^{2+}\right]}=\frac{K_{d,S}{\left[S\right]}_{tot}}{{\left(K_{d,S}+\left[Ca^{2+}\right]\right)}^{2}},$$ (1)

$$\kappa_{B}=\frac{d\left[CaB\right]}{d\left[Ca^{2+}\right]}=\frac{K_{d,B}{\left[B\right]}_{tot}}{{\left(K_{d,B}+\left[Ca^{2+}\right]\right)}^{2}},$$ (2)

where ${\left[S\right]}_{\text{tot}}$ is the total concentration of endogenous cytosolic Ca^2+^ buffers, ${\left[B\right]}_{\text{tot}}$ is the total buffer concentration including fura-2, and $K_{d,S}$ and $K_{d,B}$ are the apparent dissociation constants of the endogenous buffer and total buffer, respectively. These parameters represent the apparent behavior of the combined endogenous Ca^2+^ buffering system and do not directly measure the biochemical Ca^2+^ affinity of an individual buffer protein such as cTnC. $\kappa_{B}$ was determined from the relationship between the change in intracellular [Ca^2+^] ([Ca^2+^]_c_) and the net Ca^2+^ influx during depolarization. The Cs-pipette solution used for the buffering measurements contained 4 mM MgATP and 1 mM MgCl_2_ and was applied identically to all experimental groups (Table 1). Free cytosolic Mg^2+^ was not directly measured or independently clamped, and Mg^2+^-dependent occupancy of endogenous binding sites was not included as a separate component in the fitting model. Therefore, possible Mg^2+^-dependent effects are incorporated into the apparent parameters.

The integrated Ca^2+^ influx was divided by the accessible cell volume (${\text{vol}}_{\text{ac}}$) to obtain the calculated net increase in [Ca^2+^] ($\Delta {\left[{\text{Ca}}^{2+}\right]}_{\text{tot},\text{cal}}$), as described in our previous work (Jeon et al., 2022). After establishing the whole-cell configuration, cells were depolarized with a ramp pulse from −70 to −40 mV (300 ms) to inactivate voltage-gated Na^+^ channels, followed by a 100-ms step to +10 mV to activate $I_{\text{Ca},L}$. After confirming stable Ca^2+^ transients in NT solution, cells were treated with the SERCA inhibitor thapsigargin (2.5 µM, 5 min), followed by 20 mM caffeine to deplete SR Ca^2+^ stores. The extracellular solution was then switched to 0Ca/0Na solution (Table 1) to block Na^+^/Ca^2+^ exchanger (NCX). Subsequently, Ca^2+^ was reintroduced (0Na/2Ca; Table 1) while applying repetitive depolarizing pulses from −40 to +10 mV (100 ms, 1 Hz). The change in ${\left[{\text{Ca}}^{2+}\right]}_{c}$ ($d{\left[{\text{Ca}}^{2+}\right]}_{c}$) during each pulse was measured by fura-2 ratiometry at room temperature, and the corresponding change in bound Ca^2+^ (d[BCa]) was calculated using Eq. 3 to derive $\kappa_{B}$.

$$d\left[BCa\right]=\Delta {\left[{\text{Ca}}^{2+}\right]}_{\text{tot},\text{cal}}-d{\left[Ca^{2+}\right]}_{c}.$$ (3)

### *In situ* calibration of fura-2 fluorescence ratiometry

Accurate calibration of intracellular Ca^2+^ concentration in cardiomyocytes is technically challenging because cells undergo hypercontraction at ${\left[{\text{Ca}}^{2+}\right]}_{c}$ above ∼1 µM, which alters optical path length and can compromise membrane integrity (Cheung et al., 1989). To circumvent these limitations, we determined the calibration parameters (R_min_, R_max_, and β) in separate experiments using the lanthanum (La^3+^) method, which allows estimation of calibration constants without exposing intact cells to high Ca^2+^ levels (Borzak et al., 1990), as described in our previous work (Jeon et al., 2022). Fluorescence conversion factors were obtained using a cuvette-based spectrophotometer with an identical excitation source and were calculated using the same values as in our previous study.

To estimate the effective dissociation constant of fura-2 in the cytosolic environment, we performed an *in vitro* calibration following Berlin et al. (1994) and our previous work. Among standard, viscosity-corrected (sucrose), and protein-containing calibration solutions, the protein-containing condition most closely reflected the intracellular environment. Accordingly, and in line with our previous work (Jeon et al., 2022), we adopted the previously reported calculated value of $K_{d,\text{fura}-2}$ = 381.8 nM for subsequent analyses.

Total cellular volume (${\text{vol}}_{\text{tc}}$) was estimated from membrane capacitance ($C_{m}$), assuming a $C_{m}/{\text{vol}}_{\text{tc}}$ ratio of 6.76 pF·pl^−1^ for 12-wk-old rats (Satoh et al., 1996). Because mitochondria and other organelles occupy ∼35% of cardiomyocyte volume, the fura-2–accessible cytosolic volume (${\text{vol}}_{\text{ac}}$) was taken as 0.65·${\text{vol}}_{\text{tc}}$ (Berlin et al., 1994). To estimate the effective cytosolic concentration of fura-2 ([fura-2]_tot_), we measured fluorescence at the isosbestic wavelength at the end of each experiment. Cellular fluorescence ($F_{\text{cell}}$) was normalized to ${\text{vol}}_{\text{ac}}$ and compared with a standard fluorescence ($F_{\text{standard}}$) obtained from a glass capillary (20-µm inner diameter) filled with 50 µM fura-2 positioned in the same region of interest. [fura-2]_tot_ was calculated as described in our previous work (Jeon et al., 2022).

### Solution and chemicals

Solutions for electrophysiology, Ca^2+^ measurement, and the IonOptix system are listed in Table 1. We used four external solutions: K-B, NT, 0Na/0Ca, and 0Na/2Ca. Three types of pipette solutions were used depending on the experimental conditions (Cs-pipette and *I*_Ca,L_-pipette). Unless otherwise specified, all drugs and chemicals were purchased from Sigma-Aldrich.

### Randomization and blinding

Animals were randomly assigned to experimental groups where applicable. Blinding was not feasible for experiments requiring drug treatment during data acquisition, as treatment conditions were evident to the experimenter. In contrast, immunoblotting and NO measurements were performed with investigators blinded to group identity. Data processing and quantitative analyses were also conducted in a blinded manner whenever feasible.

### Statistical analysis and data presentation

Data are presented as mean ± standard deviation (SD), with *n* indicating the number of cells and *N* indicating the number of animals. Statistical comparisons were performed using two-tailed *t* tests or two-tailed nested *t* tests, where appropriate, to account for the hierarchical structure of the data, with cells nested within animals (*n* cells within *N* animals) (Sikkel et al., 2017; Eisner, 2021). Differences in *I*_Ca,L_ current amplitudes were evaluated using multivariate ANOVA (MANOVA). To directly compare treatment effects between chambers, cell-level measurements were averaged within each animal and condition, and data were analyzed using two-way repeated-measures ANOVA with chamber and treatment. Differences in treatment responses between chambers were determined from the chamber-by-treatment interaction. Statistical significance was accepted at P < 0.05. Exact P values are reported for statistically significant comparisons in the figure or figure legend, except for values below 0.0001, which are reported as P < 0.0001. Curve fitting was performed using a least-squares method in Origin (Microcal Software) or custom-written R scripts.

### Online supplemental material

**Figure S1.**
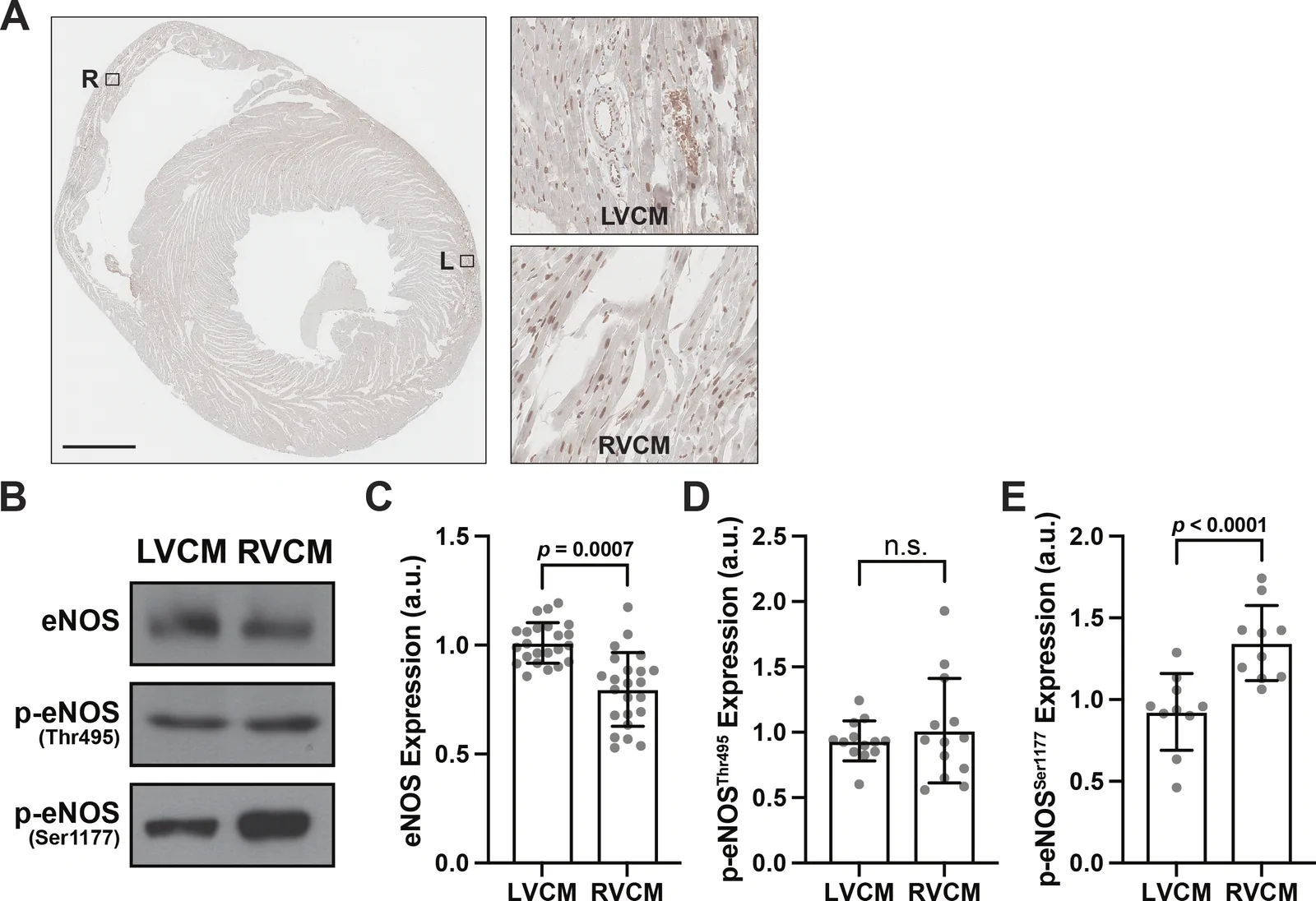
**Differential phosphorylation of eNOS in LVCM and RVCM. (A)** Representative immunohistochemistry showing nNOS staining in transverse sections of adult rat hearts. Low-magnification images indicate the regions of the LV and RV used for higher-magnification views. Enlarged images illustrate representative staining patterns in LVCM and RVCM (scale bar = 2 mm). **(B)** Representative immunoblots showing total eNOS, phosphorylated eNOS at Thr495 (p-eNOS^Thr495^), and phosphorylated eNOS at Ser1177 (p-eNOS^Ser1177^) in LVCM and RVCM. **(C)** Quantification of total eNOS expression. eNOS protein levels were significantly lower in RVCM compared with LVCM. **(D)** Quantification of p-eNOS^Thr495^ expression. No significant difference was observed between LVCM and RVCM. **(E)** Quantification of p-eNOS^Ser1177^ expression. Phosphorylation at Ser1177 was significantly increased in RVCM relative to LVCM. Each dot represents one biological sample from an individual animal, and one sample per animal per condition was included in the quantitative analysis (*N* = 16 animals). Source data are available for this figure: SourceData FS1.

**Figure S2.**
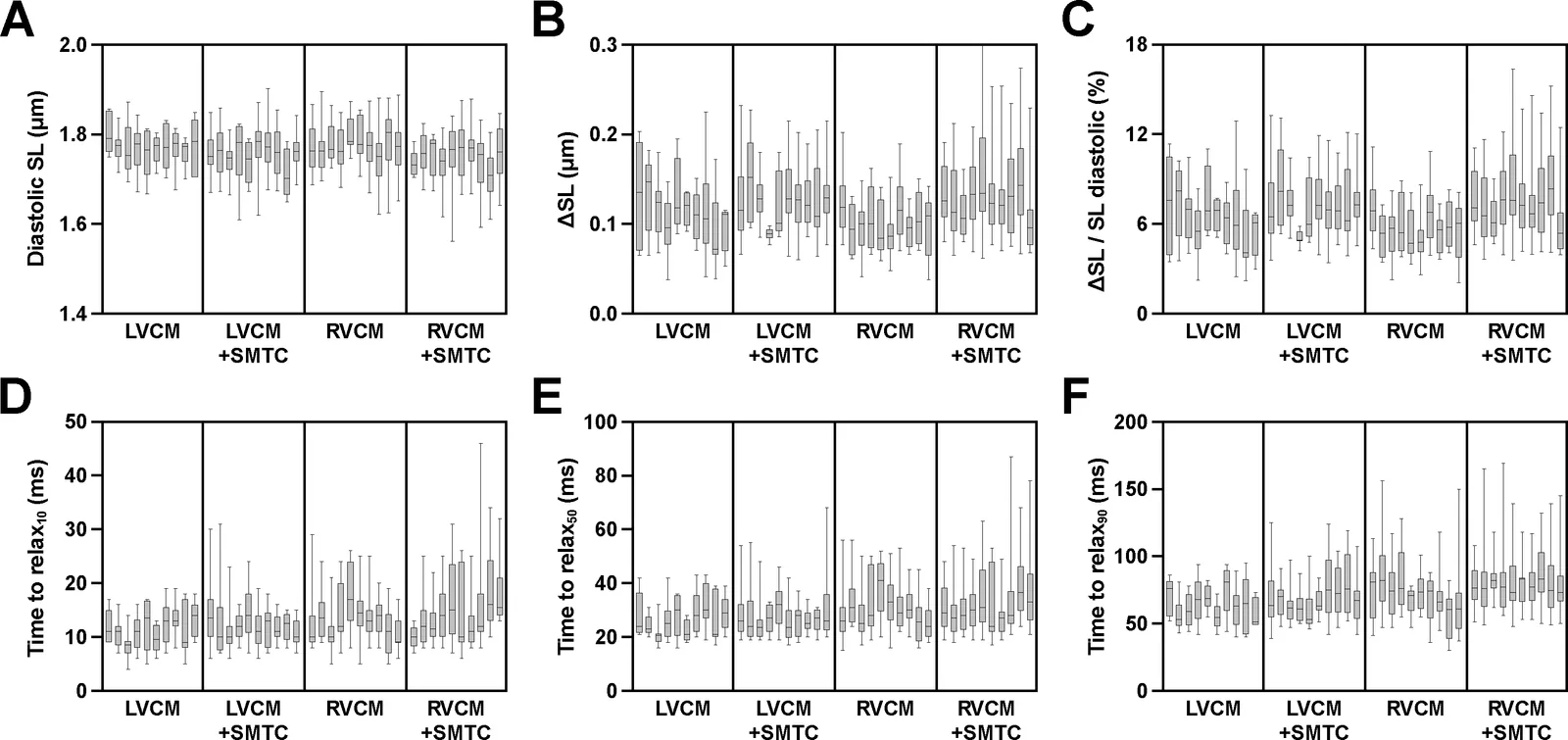
**Distribution of sarcomere shortening and relaxation parameters in LVCM and RVCM with or without SMTC. (A)** Distribution of diastolic SL. **(B)** Distribution of sarcomere shortening amplitude (ΔSL). **(C)** Distribution of fractional sarcomere shortening (ΔSL/diastolic SL). **(D)** Time to 10% relaxation. **(E)** Time to 50% relaxation. **(F)** Time to 90% relaxation. Data are shown as box-and-whisker plots representing cell-level measurements from LVCM, LVCM + SMTC, RVCM, and RVCM + SMTC. Boxes indicate the interquartile range with the median shown as a horizontal line; whiskers denote the minimum and maximum values. Each box represents the distribution of values obtained from individual cardiomyocytes pooled across animals.

**Figure S3.**
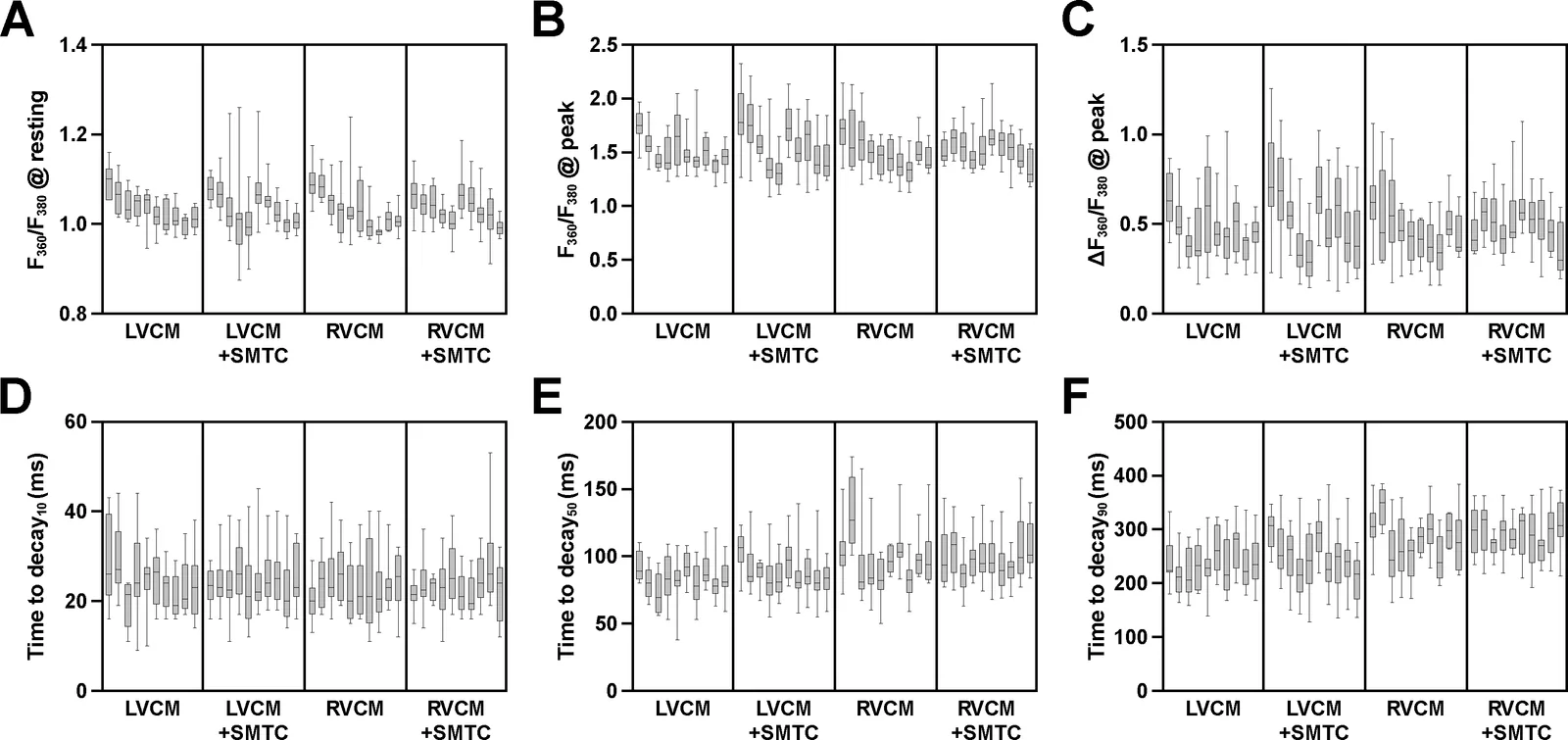
**Cell-level distributions of Ca** ^**2+**^ **transient parameters in LVCM and RVCM with or without SMTC. (A)** Resting Ca^2+^ level expressed as F_360_/F_380_ at rest. **(B)** Peak Ca^2+^ signal (F_360_/F_380_ at peak). **(C)** Ca^2+^ transient amplitude (F_360_/F_380_ at peak). **(D)** Time to 10% decay of the Ca^2+^ transient. **(E)** Time to 50% decay of the Ca^2+^ transient. **(F)** Time to 90% decay of the Ca^2+^ transient. Data are presented as box-and-whisker plots showing cell-level measurements from LVCM, LVCM + SMTC, RVCM, and RVCM + SMTC. Boxes represent the interquartile range with the median indicated by a horizontal line; whiskers denote the minimum and maximum values. Each box reflects the distribution of Ca^2+^ transient parameters obtained from individual cardiomyocytes pooled across animals.

**Figure S4.**
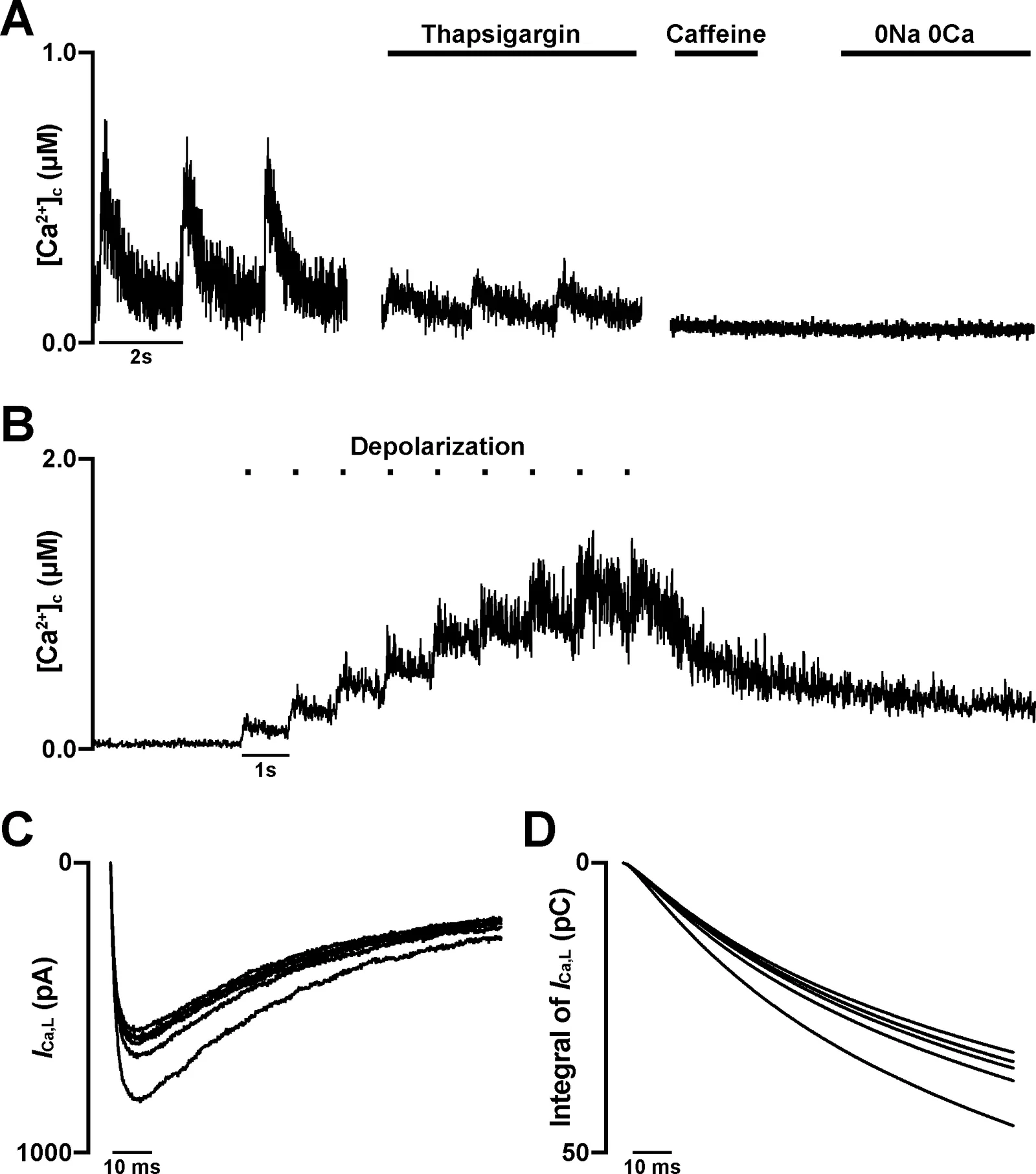
**Representative experimental traces used for intracellular Ca** ^**2+**^ **buffer analysis. (A)** Representative cytosolic Ca^2+^ concentration ([Ca^2+^]_c_) trace obtained from a control LVCM during sequential application of thapsigargin, caffeine, and a Na^+^- and Ca^2+^-free solution. After confirming Ca^2+^ transients evoked by depolarizing steps from −70 to 0 mV for 200 ms, SERCA was inhibited with thapsigargin while stimulation was continued. Caffeine (20 mM) was then applied to confirm depletion of SR Ca^2+^ stores. Finally, the normal Tyrode solution was replaced with 0Na^+^/0Ca^2+^ solution to inhibit NCX-mediated Ca^2+^ transport. **(B)** Representative ${\left[{\text{Ca}}^{2+}\right]}_{c}$ response to a series of membrane depolarizations used to determine the relationship between Ca^2+^ influx and the corresponding increase in cytosolic Ca^2+^ concentration ($\Delta {\left[{\text{Ca}}^{2+}\right]}_{c}$). Dots indicate 100-ms depolarizing pulses. After a steady state was reached, depolarizing pulses from −40 to 0 mV were applied at 1 Hz for 6–8 repetitions to evoke inward Ca^2+^ currents. **(C)** Representative inward Ca^2+^ currents evoked by the depolarizing pulses shown in B. **(D)** Time integrals of the inward Ca^2+^ currents, expressed as the quantity of electrical charge and used to estimate cumulative Ca^2+^ entry for calculation of the Ca^2+^ binding ratio, as described in the Materials and methods. These traces represent an individual experiment underlying the buffer analysis presented in Fig. 6.

Fig. S1 presents immunohistochemical analyses showing the expression levels and relative ratios of eNOS and phospho-eNOS in the cytosolic fraction, as well as the expression patterns of nNOS in RVCMs and LVCMs. Fig. S2 shows the sarcomere shortening analysis for each individual animal, and Fig. S3 shows the Ca^2+^ transient analysis for each individual animal. The box-and-whisker plots shown in Figs. S2 and S3 display the five-number summary of the data distribution, including the minimum, first quartile, median, third quartile, and maximum. Fig. S4 provides a representative example of the experimental traces used for buffer analysis, illustrating how the data presented in Fig. 6 were obtained.

## Results

### Similar expression of nNOS in the membrane/cytoplasmic fraction of LVCMs and RVCMs

Immunoblot analysis of the membrane/cytosol fraction revealed two distinct immunoreactive bands corresponding to nNOS isoforms: a lower band below 140 kDa representing the β-variant (nNOSβ) and a higher band above 165 kDa corresponding to α/μ isoforms (nNOSα/μ), consistent with previously reported isoform-specific migration patterns (Jang et al., 2015). In this study, we quantified cytosolic nNOS using the band corresponding to the nNOSα form, whereas nNOS in the myofilament fraction was assessed using the nNOSβ band (Jang et al., 2015).

**Figure 1.**
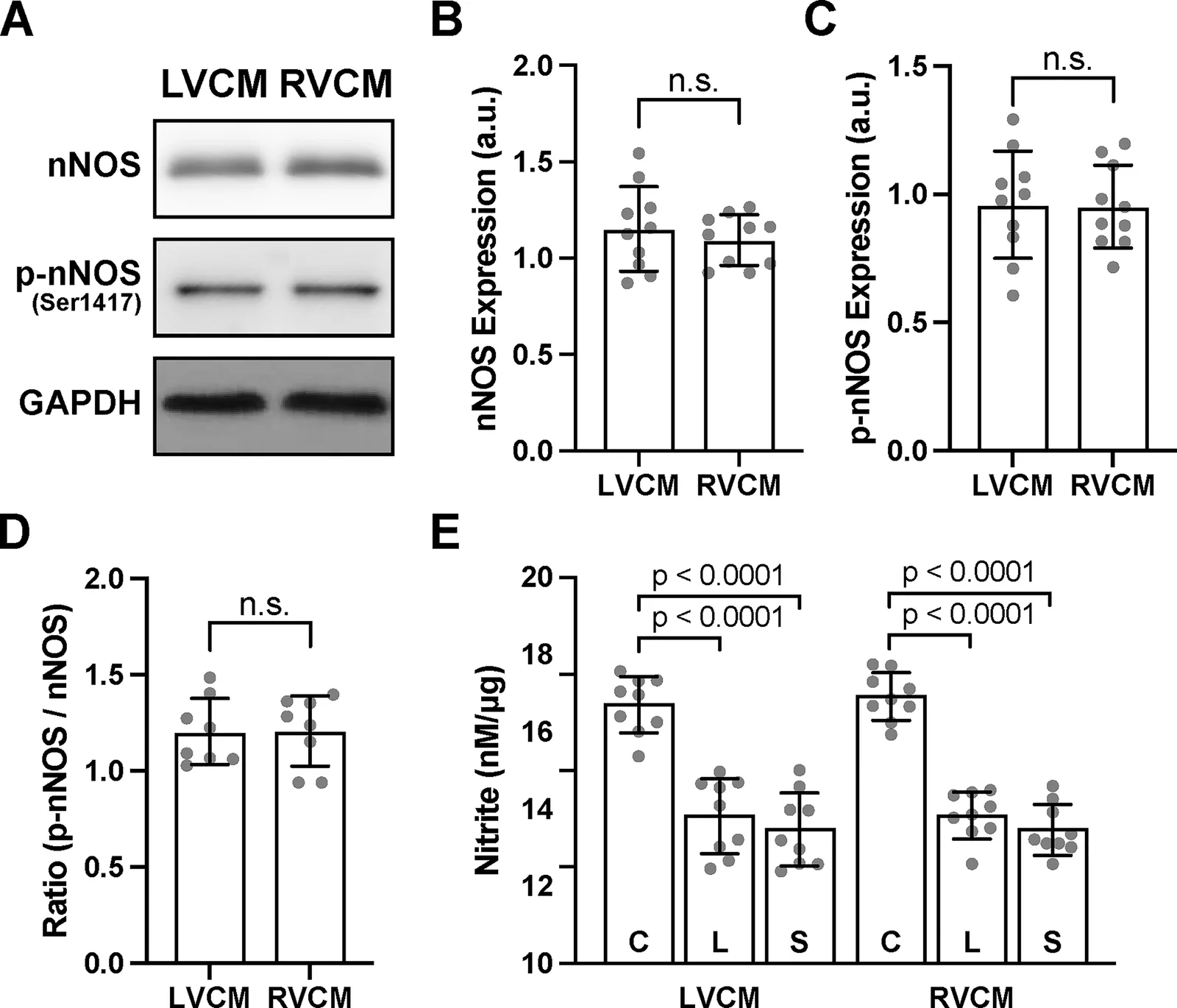
**Similar levels of total nNOS expression and NO production between LVCMs and RVCMs. (A)** Representative immunoblots showing total nNOS, p-nNOS (p-nNOS^Ser1417^), and GAPDH in LVCMs and RVCMs. **(B)** Quantification of total nNOS expression normalized to GAPDH in LVCMs (*N* = 10) and RVCMs (*N* = 10). **(C)** Quantification of p-nNOS^Ser1417^ expression normalized to GAPDH in LVCMs and RVCMs. **(D)** Ratio of p-nNOS^Ser1417^ to total nNOS in LVCMs and RVCMs. No significant differences were detected between groups. **(E)** Nitrite content measured in LVCMs and RVCMs under control conditions (C) and following inhibition of nNOS with L-NAME (L) or SMTC (S). Both inhibitors significantly reduced nitrite levels in LVCMs and RVCMs compared with control, with no significant difference between ventricular cell types (*N* = 9). Each dot represents one biological sample from an individual animal, and one sample per animal per condition was included in the quantitative analysis. n.s., not significant. Source data are available for this figure: SourceData F1.

Quantitative densitometric analysis revealed no significant difference in total nNOS protein expression between LVCMs and RVCMs (Fig. 1, A and B). The subcellular distribution of nNOS was also comparable between LVCMs and RVCMs, exhibiting a diffuse cytosolic pattern with relatively dense nuclear signal in both populations (Fig. S1 A). We further assessed the phosphorylation status of nNOS at serine 1417 (p-Ser^1417^), a well-characterized regulatory site known to enhance electron transfer from the reductase to the oxygenase domain and thereby increase catalytic efficiency (Andrabi et al., 2023) (Fig. 1, C and D). Neither p-Ser^1417^ levels nor the ratio of phosphorylated nNOS (p-nNOS) to total nNOS differed significantly between the two groups.

While nNOS plays a more direct role in regulating contractile function, eNOS is also known to participate in cardiomyocyte NO signaling. Therefore, we examined eNOS levels to distinguish the contributions of these isoforms (Tang et al., 2014). Accordingly, we analyzed both total eNOS and its phosphorylated forms (Fig. S1, B–E). In the RVCM cytosolic fraction, total eNOS expression was modestly lower, whereas phosphorylation at Ser^1177^, one of the two major activating sites, was slightly higher than that of LVCMs (Tang et al., 2014). This reciprocal change suggests that the elevated phosphorylation likely compensates for the lower protein abundance, maintaining overall eNOS activity at levels comparable with LVCMs - a conclusion further supported by our pharmacological inhibition experiments (Fig. 1 E).

To directly quantify and compare intracellular NO levels between LVCMs and RVCMs, we performed the Griess assay (Fig. 1 E). Under basal conditions, NO production was comparable between the two groups. To dissect the relative contributions of NOS isoforms, we employed pharmacological inhibitors: the nonselective NOS inhibitor N-omega-Nitro-L-arginine methyl ester hydrochloride (L-NAME, 100 μM), and the nNOS-selective inhibitor SMTC (100 nM). Treatment with L-NAME markedly reduced NO levels to a similar extent in both LVCMs and RVCMs. Similarly, SMTC caused a significant and comparable reduction in NO production in both populations. These findings suggest that global nNOS-derived NO generation is functionally similar between LVCMs and RVCMs. Furthermore, the observation that nNOS inhibition largely recapitulated the effects of nonselective blockade implies that eNOS contributes minimally to the total cytosolic NO pool, likely exerting its effects in a compartmentalized manner. Consequently, SMTC was selected as the specific nNOS inhibitor for subsequent experiments.

### Effects of nNOS inhibition on the contraction–relaxation of RVCMs and LVCMs

**Figure 2.**
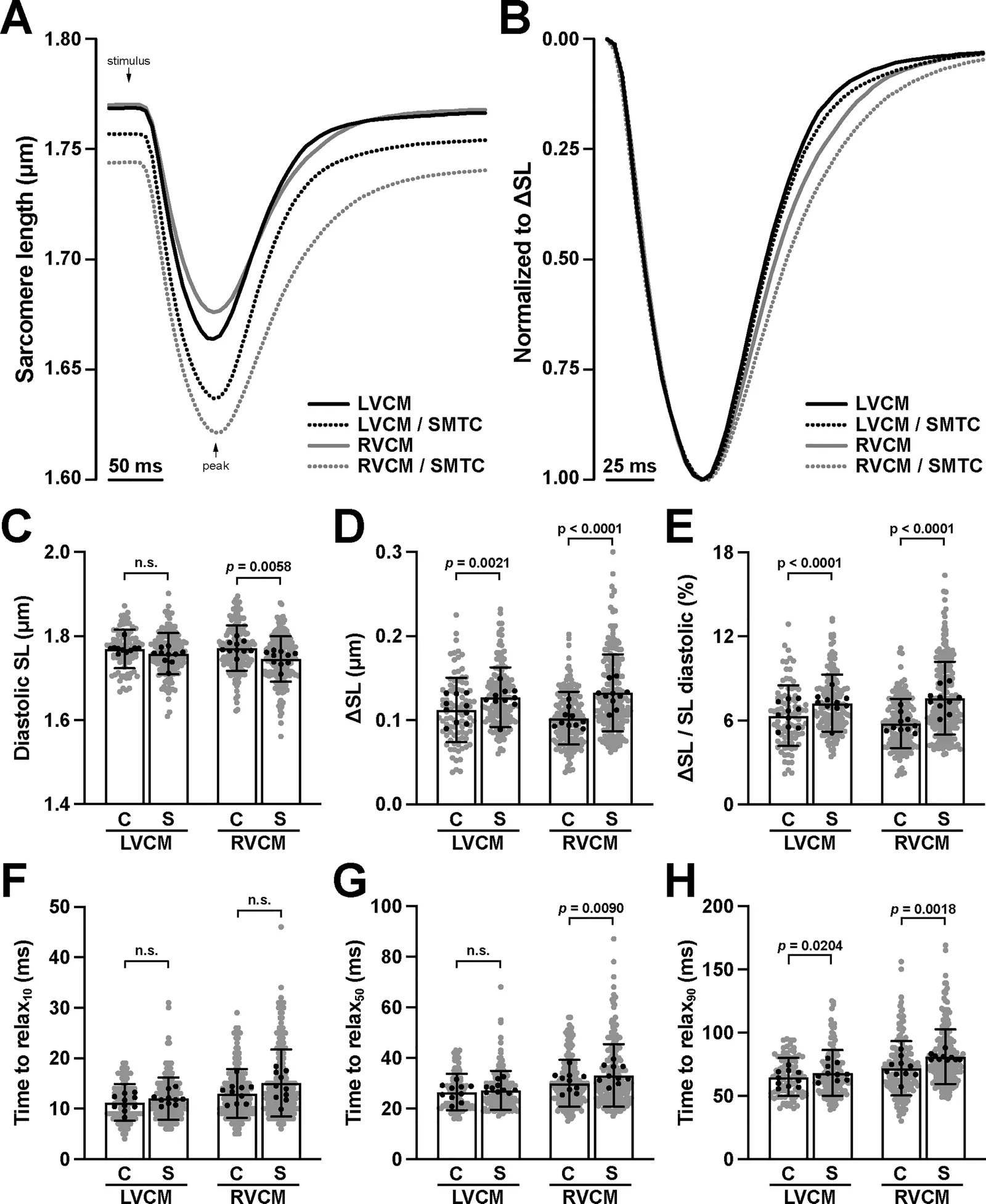
**nNOS inhibition enhances sarcomere shortening and delays relaxation more prominently in RVCMs. (A)** Representative SL traces from LVCMs and RVCMs under control conditions and following nNOS inhibition with SMTC. The timing of electrical stimulation and peak shortening are indicated. Solid black line, LVCM control; black dotted line, LVCM + SMTC; solid gray line, RVCM control; gray dotted line, RVCM + SMTC. The timing of electrical stimulation and peak shortening are indicated. **(B)** SL traces from panel A normalized to the amplitude of sarcomere shortening (ΔSL) to compare the time course of shortening and relaxation across conditions. **(C)** Diastolic SL measured in LVCMs and RVCMs under control (C) and SMTC-treated (S) conditions. **(D)** Absolute sarcomere shortening (ΔSL) in LVCMs and RVCMs under control and SMTC-treated conditions. **(E)** Sarcomere shortening normalized to diastolic SL (ΔSL/diastolic SL). **(F–H)** Time to 10% (F), 50% (G), and 90% (H) relengthening following peak sarcomere shortening in LVCMs and RVCMs under control and SMTC-treated conditions. In panels C–H, gray dots represent individual cardiomyocytes, whereas black dots represent the mean value for each animal. Sample sizes were as follows (*N* = 10 animals per group): LVCM, *n* = 92; LVCM + SMTC, *n* = 152; RVCM, *n* = 165; RVCM + SMTC, *n* = 209. n.s., not significant.

Using the IonOptix system (1 kHz sampling), we assessed SL dynamics in cardiomyocytes paced at 2 Hz via EFS. Fig. 2, A and B, display the averaged SL traces synchronized to EFS and their normalized counterparts, respectively. We analyzed the extent of sarcomere shortening (ΔSL; diastolic SL minus minimal SL) and post-contraction relaxation kinetics (Fig. 2, C–H; individual-animal data are shown in Fig. S2).

While SMTC treatment significantly increased ΔSL in both LVCMs and RVCMs (Fig. 2, D and E), the SMTC-induced increase in mean ΔSL was significantly greater in RVCMs (0.030 µm) than in LVCMs (0.015 µm; Fig. 2 D). To directly compare the magnitude of the SMTC response between chambers, the resulting animal-level means were analyzed using two-way repeated-measures ANOVA with chamber and treatment. The significant chamber-by-treatment interaction (*F*_1,9_ = 24.13, P = 0.0008) demonstrated that the SMTC-induced increase in ΔSL was greater in RVCMs. Interestingly, nNOS inhibition significantly reduced diastolic SL in RVCMs, whereas this reduction was not statistically significant in LVCMs (Fig. 2, A and C). After SMTC treatment, RVCMs exhibited a significantly shorter diastolic SL (1.74 ± 0.035 µm; Fig. 2 C), while the corresponding value in LVCMs (1.76 ± 0.021 µm) did not differ significantly. A significant chamber-by-treatment interaction was also observed for diastolic SL (*F*_1,9_ = 6.15, P = 0.0349), indicating a greater reduction in RVCMs. As a result, the increase in fractional shortening (expressed as percentage of diastolic SL) was markedly higher in RVCMs than in LVCMs, indicating a preferential inotropic response in the RV (Fig. 2 E).

Given the established lusitropic role of nNOS in cardiomyocytes (Zhang et al., 2014), inhibition of nNOS was expected to slow the relaxation following EFS-induced contraction. To compare the relaxation kinetics, SL traces were normalized (scale 0–1). These normalized averaged traces clearly demonstrated a markedly greater slowing of relaxation in SMTC-treated RVCMs compared with LVCMs (Fig. 2 B). We quantified the time to 10%, 50%, and 90% relaxation, reflecting the early, mid, and late phases, respectively (Fig. 2, F–H). SMTC treatment prolonged 90% relaxation time in both cell types, while the time to 10% relaxation remained unaltered. The lack of effect on the early phase likely reflects its dependence on cytosolic Ca^2+^ decay, whereas mid-to-late phases are more influenced by myofilament crossbridge kinetics (Biesiadecki et al., 2014). Consistent with this, SMTC treatment revealed a more robust lusitropic effect of nNOS in the mid-to-late phases in RVCMs. Although the time to 90% relaxation was increased in both groups, RVCMs exhibited a markedly larger delay (mean increase: 9.27 ms) than LVCMs (mean increase: 3.28 ms; Fig. 2 H). Although the numerical increase was greater in RVCMs than in LVCMs, the chamber-by-treatment interaction was not statistically significant (*F*_1,9_ = 2.80, P = 0.1284). These findings indicate that nNOS inhibition impairs relaxation in both ventricles.

### Chamber-specific effects of nNOS inhibition on Ca^2+^ handling

**Figure 3.**
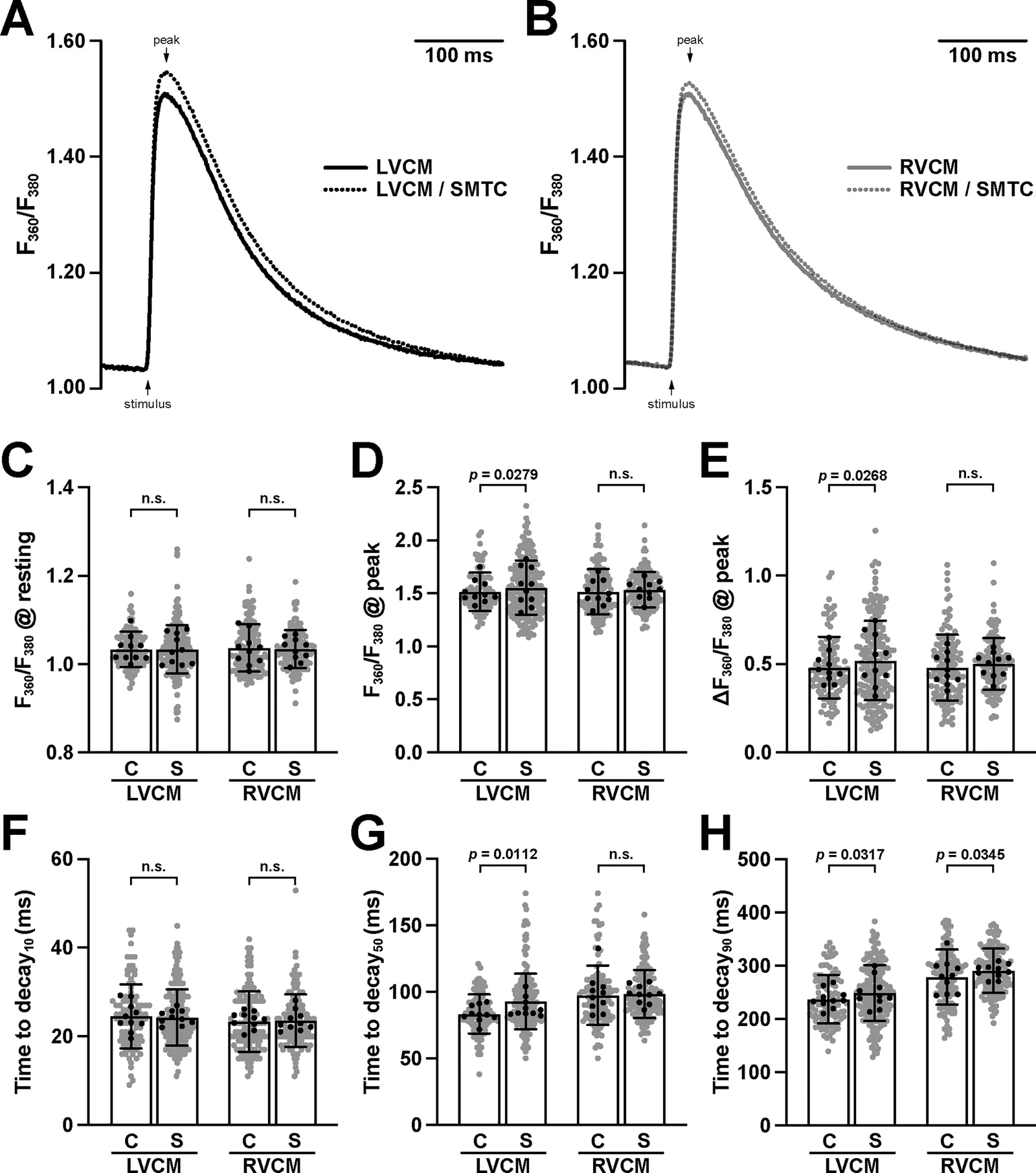
**Effects of nNOS inhibition on Ca** ^**2+**^ **transients in LVCMs and RVCMs. (A)** Representative intracellular Ca^2+^ transient traces recorded as F_360_/F_380_ in LVCMs under control conditions and following inhibition of nNOS with SMTC. Solid black line, LVCM; black dotted line, LVCM + SMTC. The timing of electrical stimulation and peak Ca^2+^ signal are indicated. **(B)** Representative Ca^2+^ transient traces from RVCMs under control conditions and following SMTC treatment. Solid gray line, RVCM; gray dotted line, RVCM + SMTC. **(C)** Resting Ca^2+^ signal expressed as F_360_/F_380_ was measured. **(D)** Peak Ca^2+^ signal (F_360_/F_380_ at peak) measured in each experimental group. **(E)** Ca^2+^ transient amplitude expressed as ΔF_360_/F_380_ at peak. **(F–H)** Time to 10% (F), 50% (G), and 90% (H) decay of the Ca^2+^ transient following peak. In panels C–H, gray dots represent individual cardiomyocytes, whereas black dots represent the mean value for each animal. Sample sizes were as follows (*N* = 10 animals per group): LVCM, *n* = 103; LVCM + SMTC, *n* = 201; RVCM, *n* = 127; RVCM + SMTC, *n* = 136. n.s., not significant.

To explore the mechanism underlying the chamber-specific contraction–relaxation responses, we examined intracellular Ca^2+^ transients in LVCMs and RVCMs paced at 2 Hz, using fura-2 fluorescence ratiometry (F_360_/F_380_) with an IonOptix system at a sampling frequency of 250 Hz (Fig. 3, A and B; individual-animal data are shown in Fig. S3). Basal diastolic Ca^2+^ levels (resting F_360_/F_380_) were comparable between LVCMs and RVCMs and remained unaltered by SMTC treatment (Fig. 3 C). However, nNOS inhibition significantly increased both the peak fluorescence ratio (peak F_360_/F_380_) and the amplitude of Ca^2+^ transients (ΔF_360_/F_380_) in LVCMs (*F*_1,9_ = 6.48, P = 0.0314). In contrast, these effects were modest and did not reach statistical significance in RVCMs (Fig. 3, D and E). Notably, this pattern was strikingly discordant with the SL data, where nNOS inhibition elicited a much stronger contractile enhancement in RVCMs.

A similar dissociation was evident in the decay kinetics of Ca^2+^ transients. We quantified the decay times to 10%, 50%, and 90% recovery (Fig. 3, F–H). While SMTC tended to prolong Ca^2+^ decay in both cell types, the effect was statistically significant only in LVCMs during the mid-phase (time to 50% decay), whereas RVCMs showed no significant prolongation (Fig. 3 G). Considering that the mid-phase of Ca^2+^ decay temporally overlaps with the late phase of relaxation, the observation that SMTC markedly slowed mechanical relaxation in RVCMs without a parallel slowing of Ca^2+^ decay supports the existence of chamber-specific differences in myofilament regulatory properties.

**Figure 4.**
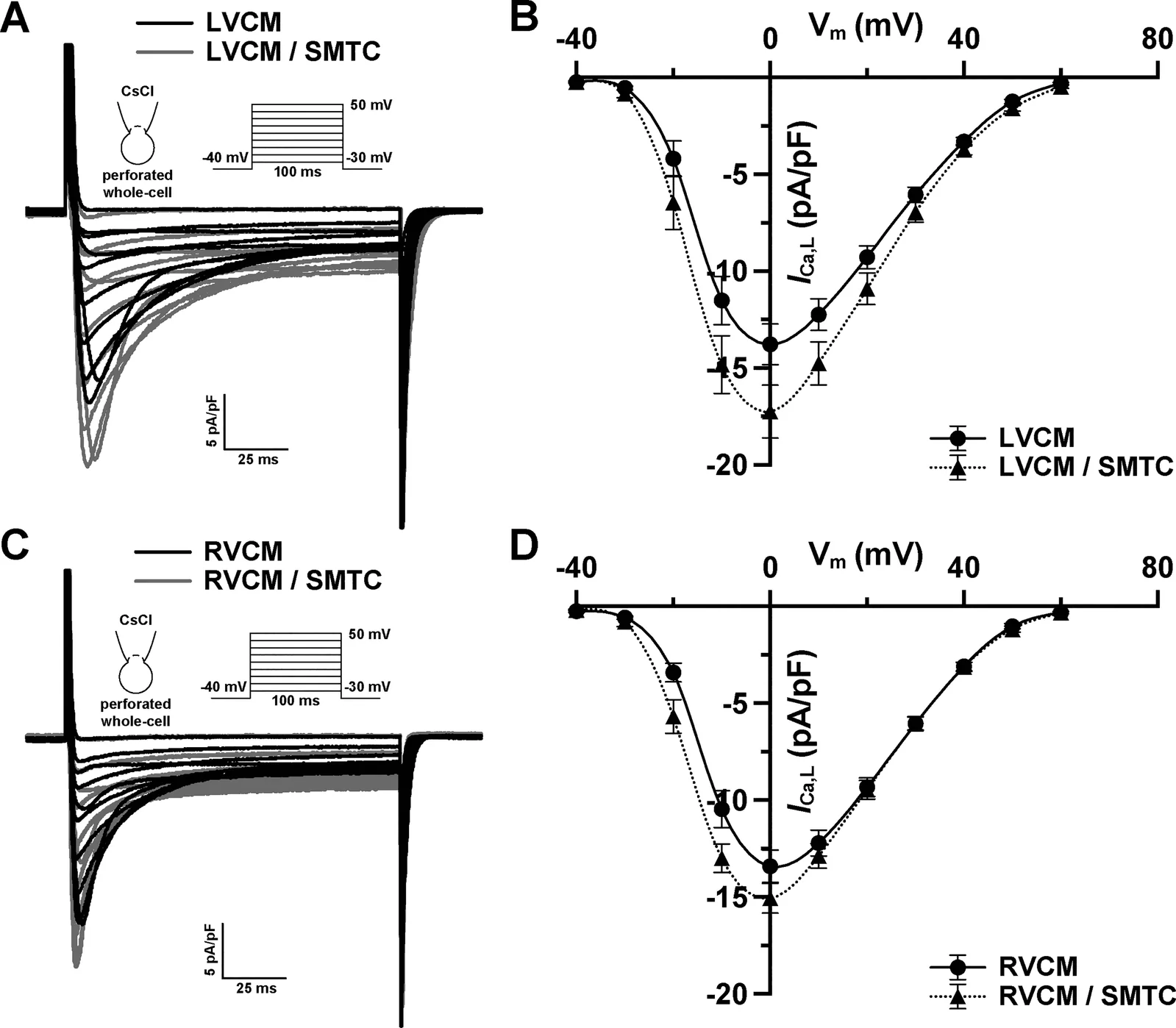
**Effects of nNOS inhibition on L-type Ca** ^**2+**^ **channel current (*I*_Ca,L_** **) in LVCM and RVCM. (A)** Representative *I*_Ca,L_ traces recorded from LVCM under control conditions and following SMTC treatment using the perforated whole-cell configuration. Solid black line, LVCM; black dotted line, LVCM + SMTC. Cells were held at −40 mV and depolarized to test potentials between −40 and +50 mV in 10 mV increments. **(B)** I-V relationships of peak *I*_Ca,L_ normalized to cell capacitance (pA/pF) in LVCM and LVCM + SMTC. Cell-level comparison of peak *I*_Ca,L_ density at the voltage producing maximal inward current demonstrated a statistically significant difference between groups (P < 0.0001). **(C)** Representative *I*_Ca,L_ traces recorded from RVCM under control conditions and following SMTC treatment using the same voltage protocol as in panel A. Solid gray line, RVCM; gray dotted line, RVCM + SMTC. **(D)** I-V relationships of peak *I*_Ca,L_ normalized to cell capacitance (pA/pF) in RVCM and RVCM + SMTC (P = 0.0008). Sample sizes were as follows: LVCM, *n* = 38 cells; LVCM + SMTC, *n* = 36 cells; RVCM, *n* = 38 cells; RVCM + SMTC, *n* = 32 cells.

Previous studies reported that nNOS-derived NO attenuates LTCCs through S-nitrosylation- or cGMP-dependent mechanisms (Sears et al., 2003). To determine if the differential Ca^2+^ transients resulted from altered Ca^2+^ entry, we recorded L-type Ca^2+^ currents (*I*_Ca,L_) using the nystatin-perforated patch-clamp technique. Step-like depolarizing voltage commands were applied from a holding potential of −40 mV. Voltage-dependent activation of inward *I*_Ca,L_ was observed (Fig. 4, A and C). Peak current amplitudes, normalized to membrane capacitance (C_m_), were plotted against test potentials to generate (I-V relationships, which did not differ between LVCMs and RVCMs under basal conditions (Fig. 4, B and D). Upon nNOS inhibition, *I*_Ca,L_ density increased in both populations; however, the magnitude of augmentation was substantially greater in LVCMs (Δ*I*_Ca,L_ = 3.4 ± 0.39 pA/pF @ 0 mV) compared with RVCMs (Δ*I*_Ca,L_ = 1.6 ± 0.49 pA/pF @ 0 mV, Fig. 4 C). Biophysical properties, including steady-state inactivation, remained unchanged (data not shown). Collectively, these findings indicate that while nNOS inhibition enhances *I*_Ca,L_-mediated Ca^2+^ influx in both chambers, the effect is blunted in RVCMs. This result provides an electrophysiological basis for the smaller augmentation of Ca^2+^ transient amplitude observed in RVCMs following nNOS inhibition, further reinforcing the paradox of their exaggerated contractile response to SMTC treatment. This mismatch between Ca^2+^ signaling and mechanical output strongly suggests an additional regulatory mechanism downstream of Ca^2+^ influx in the RVCMs.

To compare the relative magnitudes of the electrical, Ca^2+^, and mechanical responses, we calculated the percentage change relative to the corresponding control value in each chamber. In LVCMs, SMTC treatment increased peak *I*_Ca,L_ at 0 mV, Ca^2+^ transient peak amplitude, and sarcomere shortening by 25.11%, 8.31%, and 13.36%, respectively. In RVCMs, the corresponding increases were 12.30%, 4.55%, and 29.27%, respectively. Thus, although the increases in Ca^2+^ influx and Ca^2+^ transient were greater in LVCMs, the increase in sarcomere shortening was markedly greater in RVCMs. This relative mismatch between the Ca^2+^-handling and mechanical responses indicates that the enhanced contractility in RVCMs cannot be explained solely by changes in Ca^2+^ influx, suggesting an involvement of downstream myofilament regulation.

### Ca^2+^ transient-ΔSL coupling in LVCMs and RVCMs following nNOS inhibition

**Figure 5.**
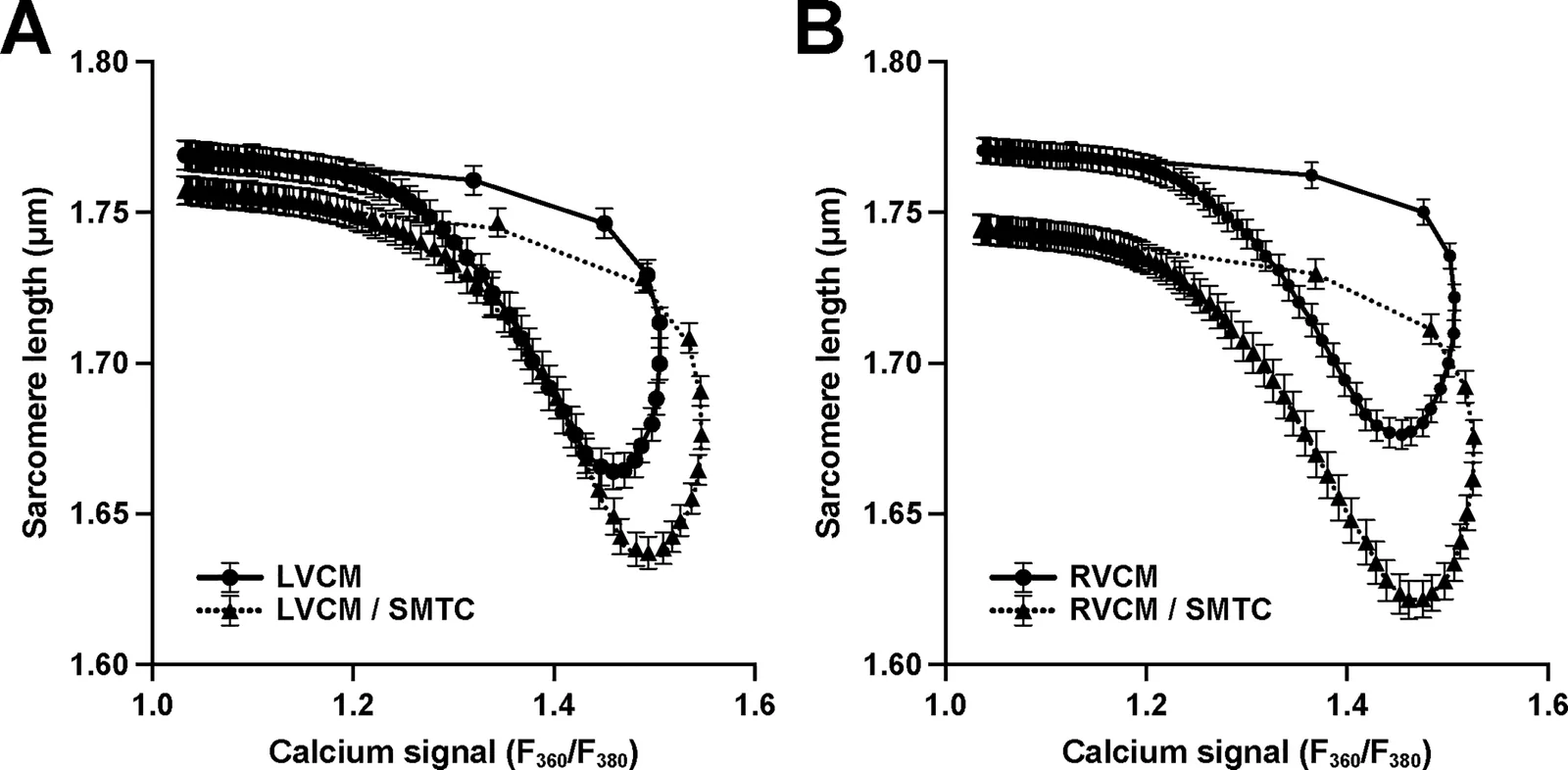
**Ca** ^**2+**^ **-sarcomere length (SL) relationship in LVCM and RVCM with or without SMTC. (A)** Cell-level phase-plane plots showing the relationship between sarcomere length and intracellular Ca^2+^ signal (F_360_/F_380_) in LVCM (solid line) and LVCM/SMTC (dotted line). Traces represent averaged trajectories constructed from individual cardiomyocytes across the full Ca^2+^ transient. **(B)** Corresponding Ca^2+^-SL relationships in RVCM (solid line) and RVCM/SMTC (dotted line).

To elucidate the mechanism underlying the dissociation between the exaggerated sarcomere shortening and the blunted Ca^2+^ transients in SMTC-treated RVCMs, we performed simultaneous measurements of SL and calcium signal (F_360_/F_380_) (Fig. 5). Plotting SL against F_360_/F_380_ over a single beat revealed characteristic hysteresis loops. In these phase-plane plots, the upper right portion of the loop corresponds to the rapid rise in intracellular Ca^2+^ during contraction, whereas the lower left portion represents the relaxation phase. The rapid rising phase is prone to distortion because force development lags behind the surge in Ca^2+^, rendering conventional steady-state measures (e.g., force-pCa) insufficient for describing dynamic behavior (Yagi et al., 1988; Chung et al., 2016). In contrast, the relaxation phase represented by the descending limb of the loop reflects a slower, quasi-equilibrium state where crossbridge detachment and myofilament properties dominate. Therefore, the slope and the horizontal position of the relaxation trajectory of the Ca^2+^–SL hysteresis loop provide a more reliable index of effective myofilament Ca^2+^ sensitivity. Accordingly, we focused our analysis on this relaxation phase.

In LVCMs, the contractile enhancement by SMTC was accompanied by a clear elevation in intracellular Ca^2+^ levels. While a modest leftward shift of the loop (indicating increased sensitivity) was observed, it did not reach statistical significance during the relaxation phase. This suggests that in LVCMs, nNOS inhibition augments contraction primarily by increasing Ca^2+^ availability rather than by altering myofilament Ca^2+^ sensitivity. Conversely, in RVCMs, nNOS inhibition resulted in a pronounced leftward shift of the relaxation trajectory (Fig. 5 B). Notably, RVCMs exhibited substantially greater shortening at equivalent Ca^2+^ levels compared with controls, and a marked reduction in SL was evident even at basal Ca^2+^ levels. Collectively, these data indicate that the augmented contraction in RVCMs is primarily attributable to a significant increase in myofilament Ca^2+^ sensitivity, with only a modest contribution from elevated intracellular Ca^2+^ levels. In contrast, in LVCMs, the enhanced contraction is driven mainly by increased intracellular Ca^2+^ concentration, with a comparatively limited contribution from changes in myofilament Ca^2+^ sensitivity.

### Quantification of endogenous Ca^2+^ buffering reveals chamber-specific modulation by nNOS

**Figure 6.**
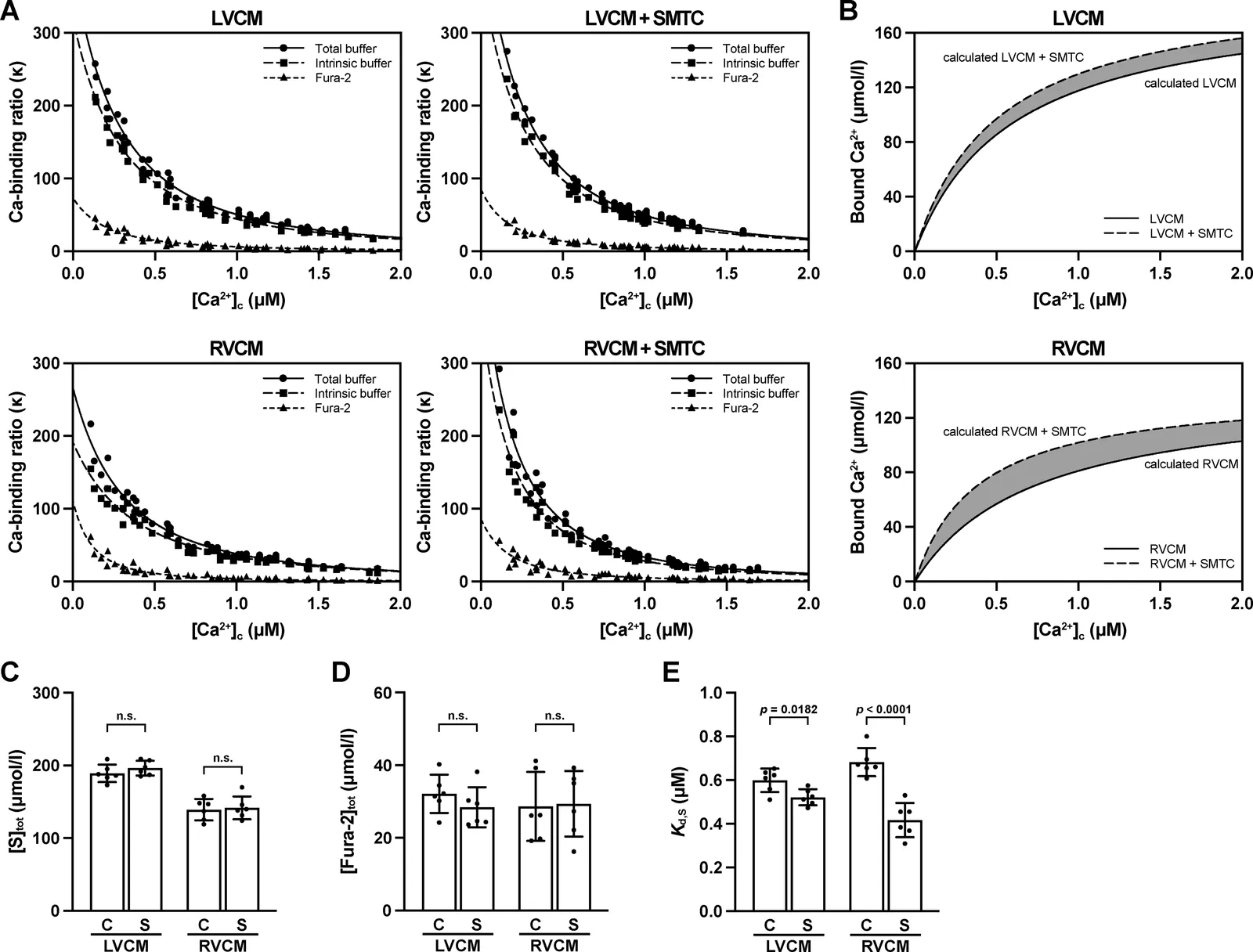
**Effects of SMTC on cytosolic Ca** ^**2+**^ **buffering properties in LVCM and RVCM. (A)** Ca^2+^-binding ratio (κ) plotted as a function of intracellular Ca^2+^ concentration (${\left[{\text{Ca}}^{2+}\right]}_{c}$) in LVCM, LVCM + SMTC, RVCM, and RVCM + SMTC. Total buffering capacity (circles), intrinsic buffering (squares), and fura-2 buffering (triangles) are shown. Lines indicate fits derived from buffering analysis (Eq. 1 and Eq. 2). **(B)** Calculated total bound Ca^2+^ as a function of [Ca^2+^]_c_ in LVCM (top) and RVCM (bottom). Solid lines indicate calculated values for control cells, and dashed lines indicate calculated values for SMTC-treated cells. Shaded areas represent the difference in bound Ca^2+^ between control and SMTC conditions across the measured Ca^2+^ range (<2 µM). **(C)** Summary of total endogenous Ca^2+^ buffer concentration ([S]_tot_) in LVCM and RVCM with (S) or without (C) SMTC. No significant differences were detected between groups. **(D)** Summary of intracellular fura-2 concentration ([fura-2]_tot_) across experimental groups. SMTC treatment did not affect indicator loading in either LVCM or RVCM. **(E)** Apparent dissociation constant ($K_{d,S}$) of endogenous Ca^2+^ buffers. SMTC significantly reduced $K_{d,S}$ in both LVCM and RVCM, with a more pronounced effect in RVCM. Individual dots represent values from independent experiments. For all groups, *N* = 6 independent experiments and *n* = 6 cells per group.

An increase in functional myofilament Ca^2+^ sensitivity may be accompanied by altered Ca^2+^-binding within the myofilament compartment, including changes involving cTnC, corresponding to a reduced dissociation constant ($K_{d}$) and, consequently, an enhanced myofilament Ca^2+^ buffering capacity as suggested by Eisner and colleagues (Eisner, 2014; Eisner et al., 2017; Eisner et al., 2023). While Ca^2+^ sensitivity cannot be attributed solely to cTnC-Ca^2+^ binding, such augmented myofilament buffering is predicted to modulate Ca^2+^ transient kinetics and exert a disproportionate influence on mechanical relaxation, even in the absence of marked changes in total intracellular Ca^2+^ levels. Because other Ca^2+^ buffers such as calmodulin and ATP contribute minimally within the physiological range of [Ca^2+^]_c_ (Smith and Eisner, 2019), we quantified the endogenous cytosolic Ca^2+^ buffering coefficient ($\kappa_{S}$) in nNOS-inhibited RVCMs and LVCMs after functionally eliminating Ca^2+^ fluxes mediated by Ca^2+^ release from SR as well as the removal by SERCA and NCX, using caffeine (20 mM), thapsigargin (2.5 µM), and a Na^+^-free bath solution, respectively (Eq. 1, Fig. 6). A detailed schematic of the experimental protocol is provided in Fig. S4.

To quantify myofilament Ca^2+^ buffering capacity and kinetics, we applied depolarizing pulse protocols in a Na^+^-free bath solution with caffeine and thapsigargin to measure cumulative Ca^2+^ entry via voltage-operated LTCCs. Changes in intracellular Ca^2+^ concentration and buffering parameters ($\kappa_{B}$ and ${\left[B\right]}_{\text{tot}}$, Eq. 1) were calculated using the added-buffer approach (Neher, 1995), as detailed in our previous study (Jeon et al., 2022). To estimate the endogenous Ca^2+^ buffer pool (${\left[S\right]}_{\text{tot}}$), the contribution of the exogenous indicator fura-2 ([fura-2]_tot_) was subtracted (Fig. 6 C). Since the dissociation constant of fura-2 (*K*_d,fura-2_) was determined via *in vitro* calibration and the effective intracellular concentration of fura-2 was established in our previous work, the relationship between the Ca^2+^-binding ratio of fura-2 (κ_fura-2_) and the median [Ca^2+^]_c_ was fitted using the differential form of the Michaelis–Menten equation (triangular symbols in Fig. 6 A). Subsequently, the endogenous buffering coefficient ($\kappa_{S}$) was derived by subtracting κ_fura-2_ from the total buffering coefficient ($\kappa_{B}$).

Fitting $\kappa_{S}$ as a function of [Ca^2+^] to Eq. 1 yielded two parameters describing the apparent properties of the endogenous Ca^2+^ buffer system: the total concentration of the endogenous buffer (${\left[S\right]}_{\text{tot}}$) and its apparent dissociation constant ($K_{d,S}$). Consistent with our previous findings demonstrating a lower myofilament content in RVCMs (Jeon et al., 2022), ${\left[S\right]}_{\text{tot}}$ was significantly lower in RVCMs than in LVCMs, and this parameter (${\left[S\right]}_{\text{tot}}$) was unaffected by SMTC treatment in either group. Intracellular fura-2 concentrations were comparable across all four groups, indicating consistent fura-2 loading (Fig. 6 D).

Although $K_{d,S}$ represents the combined apparent behavior of endogenous intracellular buffers and could not be interpreted as a direct measurement of cTnC affinity, the calculated apparent dissociation constant for the endogenous buffer ($K_{d,S}$) values (LVCMs: 0.59 ± 0.049 µM; RVCMs: 0.68 ± 0.059 µM, Fig. 6 E) were similar to the reported dissociation constant of troponin C ($K_{d,\text{cTnC}}$ <0.6 µM; Smith and Eisner [2019]). Consistent with the increase in myofilament Ca^2+^ sensitivity observed in the loop analysis, nNOS inhibition reduced $K_{d,S}$ in both ventricular cardiomyocyte types. Notably, the magnitude of this reduction was substantially greater in RVCMs ($\Delta K_{d,S}$ = −0.265 µM) than in LVCMs ($\Delta K_{d,S}$ = -0.077 µM). This indicates that nNOS inhibition produces a larger change in the apparent Ca^2+^ affinity of the endogenous buffering system in RVCMs, which may partly reflect altered Ca^2+^ binding within the cTn complex (Fig. 6 E).

Using these parameters ($\kappa_{B}$, $\kappa_{S}$, κ_fura-2_, $K_{d,B}$, $K_{d,S}$, ${\left[B\right]}_{\text{tot}}$, [fura-s]_tot_, and ${\left[S\right]}_{\text{tot}}$), we calculated the amount of Ca^2+^ bound to intracellular buffers as a function of cytosolic Ca^2+^ concentrations ([Ca^2+^]_c_) using the Michaelis–Menten equation (Fig. 6 B). In the Ca^2+^-bound Ca^2+^ relationship, solid lines represent untreated cardiomyocytes, whereas dashed lines denote SMTC-treated cells. Considering that the changes in the dissociation constant of intrinsic buffer ($K_{d,S}$) occur within the physiological range of [Ca^2+^]_c_ (<1.0 µM), the impact of nNOS inhibition on Ca^2+^ binding became more apparent within this physiological Ca^2+^ range, rather than at supraphysiological levels. Thus, nNOS inhibition produced a clearly distinguishable upward shift in bound Ca^2+^ within the physiological range in both cell types, with the effect being markedly more prominent in RVCMs.

### nNOS inhibition preferentially reduces TnI phosphorylation in RVCMs

**Figure 7.**
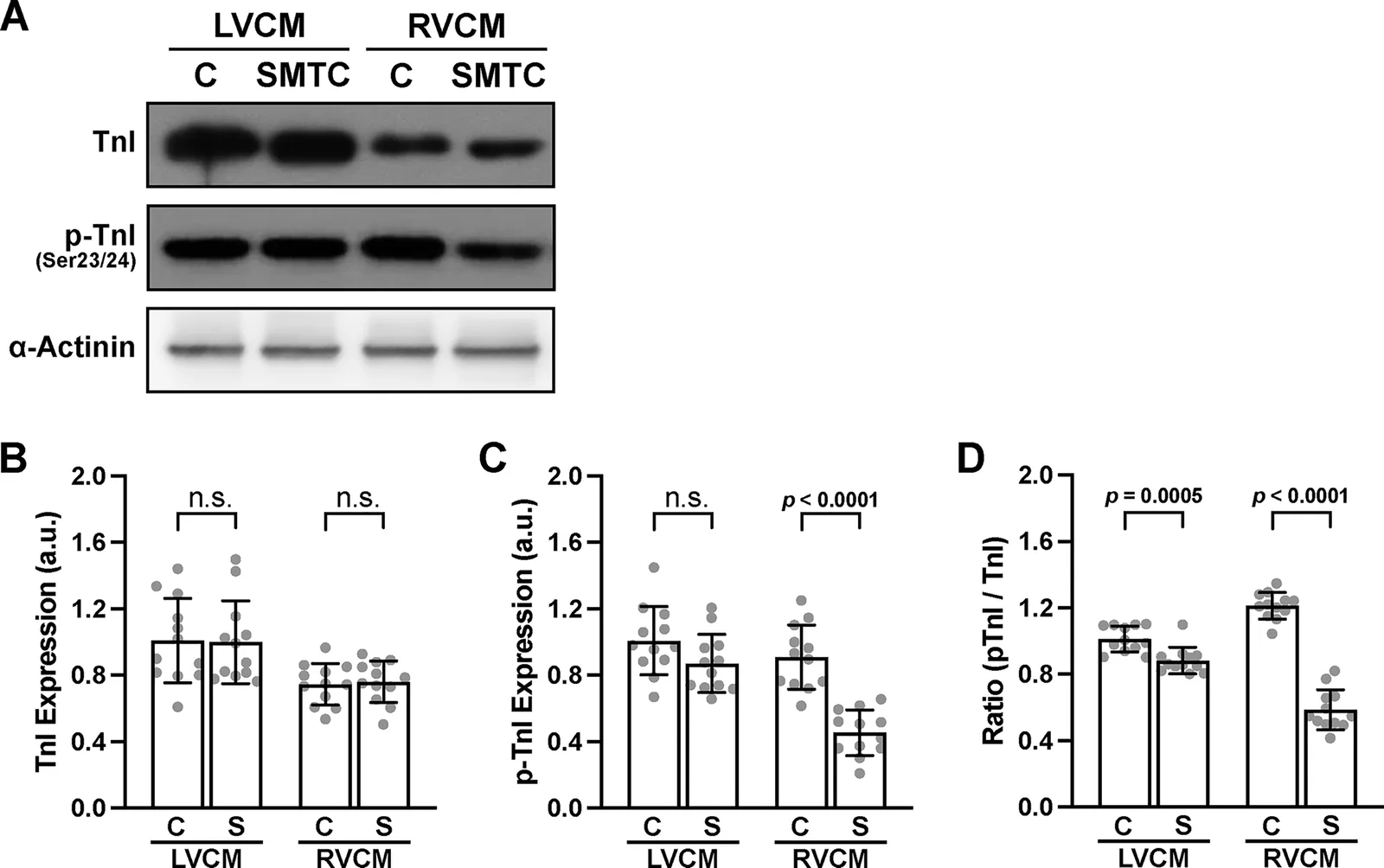
**SMTC treatment reduces TnI phosphorylation. (A)** Representative immunoblots showing total troponin I (TnI), phosphorylated TnI at Ser23/24 (p-TnI^Ser23/24^), and α-actinin in LVCM and RVCM under control conditions (C) or following SMTC treatment (S). α-Actinin was used as a loading control. **(B)** Quantification of total TnI expression normalized to α-actinin. No significant differences were observed between control and SMTC-treated cells in either LVCM or RVCM. **(C)** Quantification of p-TnI^Ser23/24^ expression normalized to α-actinin. SMTC treatment significantly reduced p-TnI levels in RVCM but not in LVCM. **(D)** Ratio of p-TnI^Ser23/24^ to total TnI. SMTC induced a modest reduction in the p-TnI/TnI ratio in LVCM and a pronounced reduction in RVCM. Each dot represents one biological sample from an individual animal, and one sample per animal per condition was included in the quantitative analysis (*N* = 12 animals). Source data are available for this figure: SourceData F7.

Since the Ca^2+^–SL loop analysis and buffering quantification pointed toward a preferential increase in myofilament Ca^2+^ sensitivity and endogenous Ca^2+^-binding properties in RVCMs, we next investigated the molecular mechanism by quantifying the phosphorylation status of cardiac cTnI, a canonical regulator of myofilament Ca^2+^ sensitivity (Fig. 7). It has been reported that p-cTnI mediated by PKA or nNOS/PKG signaling could decrease myofilament Ca^2+^ sensitivity (Zhang et al., 1995; Kooij et al., 2010; Jin et al., 2012).

In LVCMs, SMTC treatment resulted in a trend toward decreased p-cTnI, but this did not reach statistical significance. In contrast, nNOS inhibition in RVCMs elicited a marked and statistically significant reduction in p-cTnI levels (Fig. 7 C). As noted in our previous study (Jeon et al., 2022), basal total cTnI expression is lower in RVCMs than in LVCMs (Fig. 7 B). Therefore, to better assess relative differences in phosphorylation, we compared the normalized ratio of p-cTnI with total cTnI (Fig. 7 D). While SMTC significantly reduced the p-cTnI/total cTnI ratio in both groups, the magnitude of this reduction was substantially greater in RVCMs. These molecular data align closely with the functional data showing a more robust increase in Ca^2+^ sensitivity in the RV. Collectively, these findings provide a molecular explanation for the chamber-specific phenotype: nNOS inhibition preferentially reduces p-cTnI in RVCMs, providing a potential molecular basis for the greater increase in functional myofilament Ca^2+^ sensitivity and the associated change in apparent endogenous buffering properties.

### Differential myofilament-associated nNOS activation between LVCMs and RVCMs

**Figure 8.**
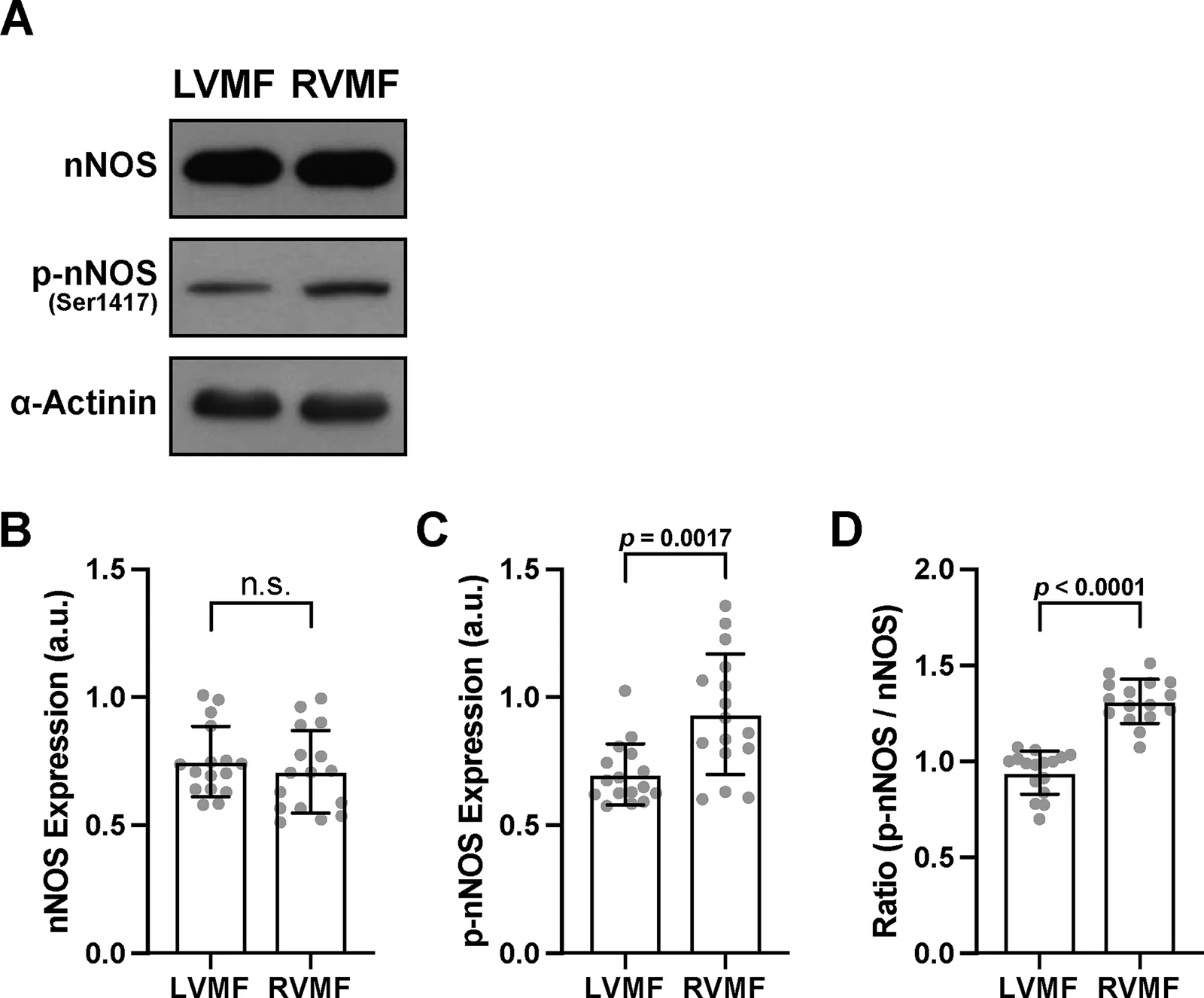
**Enhanced nNOS phosphorylation in the myofilament fraction of RVCM compared with LVCM. (A)** Representative immunoblots showing total nNOS, p-nNOS at Ser1417 (p-nNOS^Ser1417^), and α-actinin in the myofilament fractions isolated from LVMF and RVMF. α-Actinin was used as a loading control. **(B)** Quantification of total nNOS expression normalized to α-actinin in LVMF and RVMF. No significant difference was observed between groups. **(C)** Quantification of p-nNOS^Ser1417^ expression normalized to α-actinin. p-nNOS^Ser1417^ levels were significantly higher in RVMF compared with LVMF. **(D)** Ratio of p-nNOS^Ser1417^to total nNOS. The p-nNOS/nNOS ratio was markedly increased in RVMF relative to LVMF. Each dot represents one biological sample from an individual animal, and one sample per animal per condition was included in the quantitative analysis (*N* = 16 animals). Source data are available for this figure: SourceData F8.

Cardiac nNOS is known to be spatially confined to distinct subcellular compartments, including the cytosol, sarcolemma, SR, and myofilaments (Jang et al., 2015; Carnicer et al., 2017). In this study, we initially confirmed that global cellular levels of total and p-nNOS did not differ between LVCMs and RVCMs (Fig. 1). However, whole-cell lysates can mask spatial differences between functional microdomains such as myofilaments. To address this, we isolated the myofilament fraction to specifically examine the pool of nNOS (Fig. 8). While total nNOS protein levels were comparable between groups, the level of Ser^1417^ p-nNOS was markedly higher in the myofilament fraction of RVCMs, with a consequently higher p-nNOS/nNOS ratio (Fig. 8, C and D). These findings reveal a larger pool of active p-nNOS enriched within the myofilaments of RVCMs, which may help explain the greater effect of nNOS inhibition on RVCM contractility.

## Discussion

The present results indicate that nNOS signaling exerts negative inotropic and positive lusitropic effects in both LVCMs and RVCMs. However, these regulatory influences are substantially more pronounced in RVCMs, suggesting a chamber-specific functional specialization of nNOS in contraction–relaxation dynamics. Our data suggest that the myofilament Ca^2+^ sensitivity is critically regulated by the myofilament-associated pSer^1417^ nNOSβ isoform via Ser^23/24^ phosphorylation of cTnI in RVCMs.

### Compartmentalized activation of nNOSβ underlies chamber-specific regulation of E-C coupling

NO signaling in cardiomyocytes is increasingly recognized as a highly compartmentalized process rather than a diffusely acting pathway. nNOS localizes to distinct subcellular microdomains, including the SR and sarcolemma, where it spatially regulates targets involved in E-C coupling and β-adrenergic signaling (Carnicer et al., 2017). Importantly, previous studies have also demonstrated the presence of an nNOS pool within the myofilament fraction, suggesting that sarcomere proteins are subject to regulation by locally generated NO (Jang et al., 2015). Such spatial restriction provides a mechanistic framework whereby nNOS exerts selective functional effects without necessarily altering whole-cell NO levels, as downstream signaling is shaped by microdomain-specific protein interactions and local redox environments.

In the present study, although total cellular nNOS expression was comparable between LVCMs and RVCMs, we identified significantly higher levels of phosphorylated nNOSβ at Ser^1417^ within the myofilament-enriched fraction of RVCMs. Since phosphorylation at Ser^1417^ is a well-established marker of enhanced nNOS enzymatic activity (Kar et al., 2015; Andrabi et al., 2023), this finding indicates that nNOSβ activity diverges between the two chambers at the posttranslational level and is preferentially compartmentalized within the myofilament region of the RV.

Phosphorylation of the stimulatory C-terminal site of nNOS is regulated by several upstream pathways. In the intact heart, oxidative stress, mechanical cues, and β-adrenergic receptor signaling can influence nNOS phosphorylation through AMP-activated protein kinase- or Akt-dependent processes (Watts et al., 2013; Garbincius and Michele, 2015; Kar et al., 2015). Because the RV and LV experience distinct loading patterns and exhibit differences in adrenergic signaling, these inputs may contribute *in vivo* to the establishment of chamber-specific nNOSβ regulation (Redington et al., 1988; Naito et al., 1995). However, the present experiments were performed in freshly isolated, unloaded cardiomyocytes under common experimental conditions, and cells used for biochemical analyses were not maintained in culture medium. Thus, acute mechanical load or ligand-dependent receptor signaling cannot by itself account for the difference in myofilament-associated p-nNOSβ at the time of measurement. Instead, the observed difference may reflect a molecular state established *in vivo* and retained after isolation, such as a persistent local kinase–phosphatase balance, stable nNOSβ microdomain or scaffold interactions, or a pre-existing posttranslational state. The present study does not distinguish among these possibilities or establish whether the retained phenotype is developmentally programmed or a lasting consequence of the *in vivo* mechanical and neurohumoral environment.

Functionally, the enrichment of activated nNOSβ in the myofilament fraction offers a potential mechanistic explanation for the greater sensitivity of RVCMs to nNOS inhibition. Pharmacological inhibition of nNOS resulted in a more pronounced reduction in p-cTnI at Ser^23/24^ and a larger shift in the Ca^2+^-contraction relationship in RVCMs compared with LVCMs (Figs. 5 and 7). p-cTnI at Ser^23/24^ is a central determinant of myofilament Ca^2+^ sensitivity, classically mediated by PKA but also modulated by NO-dependent signaling pathways (Zhang et al., 1995; Kooij et al., 2010; Jin et al., 2012). Physiologically, the higher myofilament-associated nNOSβ in RVCMs likely promotes myofilament Ca^2+^ desensitization via local regulation of p-cTnI. This microdomain-specific organization provides a molecular basis for the chamber-dependent regulation by nNOS activity in ventricular contractile behavior.

### Chamber-specific regulation of LTCCs in response to nNOS inhibition

In addition to the chamber-dependent differences in myofilament-associated nNOS signaling, the response of LTCCs to nNOS inhibition also differed markedly between ventricular myocytes. In ventricular cardiomyocytes, nNOS has been shown to negatively regulate *I*_Ca,L_ through multiple mechanisms, including activation of the cGMP/PKG pathway and direct S-nitrosylation of LTCCs, thereby limiting Ca^2+^ influx under basal conditions (Sumii and Sperelakis, 1995; Sears et al., 2003). Consistent with this established inhibitory role of nNOS, pharmacological inhibition with SMTC resulted in a robust increase in *I*_Ca,L_ in LVCMs. In contrast, RVCMs exhibited only a modest and statistically nonsignificant increase in *I*_Ca,L_ following SMTC treatment (Fig. 4), despite comparable total nNOS expression and similar NO suppression. This suggests that nNOS-dependent regulation of LTCCs is intrinsically less prominent in RVCMs.

LTCC function is governed by an integrated network involving PKA, CaMKII, and phosphodiesterase within membrane-associated macromolecular complexes (Kamp and Hell, 2000; Bers and Grandi, 2009; Leroy et al., 2011). A previous study reported that the magnitude of *I*_Ca,L_ modulation by NOS inhibitors showed minimal changes in *I*_Ca,L_ despite detectable effects on intracellular Ca^2+^ transients (Ebner et al., 2020). Thus, variations in the composition or hierarchy of these signaling complexes could attenuate the functional impact of nNOS inhibition on *I*_Ca,L_ in the RV. While the precise molecular mechanisms remain to be elucidated, these data imply differential coupling between nNOS and LTCC regulatory pathways between LVCMs and RVCMs.

### Myofilament Ca^2+^ buffering as a determinant of apparent Ca^2+^ sensitivity

In theories of E-C coupling in cardiomyocytes, “calcium sensitivity” and “calcium buffering” are often discussed separately or interchangeably; they represent fundamentally distinct but interconnected concepts (Bers, 2002; Eisner et al., 2017; Eisner et al., 2023). Calcium sensitivity refers to the magnitude of contractile response at a given intracellular Ca^2+^ concentration, typically described by the pCa–force relationship (or, in the present study, the Ca^2+^–SL relationship). In contrast, calcium buffering describes the kinetic properties of Ca^2+^ binding and unbinding within the cell. Among the variety of binding sites, cTnC occupies a unique position as both the sensor/regulator for contraction and a major buffer modulating free Ca^2+^ availability.

When Ca^2+^ buffer affinity increases, a larger fraction of Ca^2+^ becomes bound, particularly to myofilament sites such as cTnC, resulting in a lower free cytosolic Ca^2+^ concentration ([Ca^2+^]_c_) for a given amount of Ca^2+^ entering the cytosol. This redistribution can shift the relationship between Ca^2+^ and sarcomere shortening (Ca^2+^–SL), giving the appearance of increased Ca^2+^ sensitivity in the Ca^2+^–SL relationship. Thus, increased intrinsic buffer affinity may contribute to the observed enhancement of Ca^2+^ sensitivity. We observed that pharmacological inhibition of nNOS reduced the p-cTnI significantly more in RVCMs. Since p-cTnI reduces the Ca^2+^-binding affinity of cTnC (Kentish et al., 2001; Solaro and Kobayashi, 2011), we predicted a greater increase in cTnC Ca^2+^ affinity in RVCMs following nNOS inhibition. By employing an “added-buffer approach” under conditions that minimized SERCA and NCX fluxes (Fig. 6), we could estimate the apparent properties of endogenous buffer, and observed a significantly decreased dissociation constant ($K_{d,S}$) of the endogenous Ca^2+^ buffer (binding species “S”) in both cell types (Mathias et al., 1990). The reduced $K_{d,S}$ is indicative of increased binding affinity; Ca^2+^ binds more readily at lower cytosolic concentrations. Notably, this reduction in apparent $K_{d,S}$ was quantitatively greater in RVCMs ($\Delta K_{d,S}$ = −0.265 µM vs. −0.077 µM in LVCMs), paralleling the greater reduction in p-cTnI. These findings suggest that enhanced Ca^2+^ binding to troponin may have contributed, at least in part, to the observed change in *K*_d,S_. Mg^2+^ occupancy should also be considered when interpreting $K_{d,S}$ because the C-terminal structural sites of cTnC can bind both Ca^2+^ and Mg^2+^. All groups were studied using the same MgATP- and MgCl_2_-containing pipette solution, but free intracellular Mg^2+^ was not directly measured. Thus, the absolute $K_{d,S}$ values are conditional on the Mg^2+^ environment of the whole-cell recording, and Mg^2+^-dependent effects cannot be separated from the combined apparent behavior of the endogenous buffering system.

Moreover, this effect was most pronounced within the physiological range of free Ca^2+^ concentrations (<1 µM), where differences in buffer affinity exert the greatest influence on Ca^2+^ partitioning between bound and free pools (Fig. 6 B). These results indicate that nNOS inhibition alters apparent endogenous Ca^2+^ buffering, with a greater effect in RVCMs. The parallel reduction in p-cTnI is consistent with a contribution from altered myofilament Ca^2+^ regulation (Fig. 7). This chamber-specific response is consistent with a greater contribution of myofilament-localized nNOSβ in RV (Fig. 8).

The majority of intracellular Ca^2+^ in cardiomyocytes resides in a buffer-bound state (Bers, 2002), and alterations in buffer affinity are expected to influence Ca^2+^ transient kinetics (Smith and Eisner, 2019). From this perspective, the slowing of Ca^2+^ decay observed following nNOS inhibition is consistent with an increase in Ca^2+^ buffer affinity. Notably, the effect on mechanical relaxation was substantially greater than the change in Ca^2+^ decay in RVCMs (Fig. 2 vs. Fig. 3), suggesting that altered endogenous Ca^2+^ buffering, potentially including changes at myofilament binding sites such as TnC, may contribute to the delayed mechanical relaxation. This interpretation is supported by the concept that Ca^2+^ transient kinetics reflect coordinated interactions between Ca^2+^ removal systems and intracellular buffering processes (Briston et al., 2014). Thus, altered endogenous Ca^2+^ buffering, potentially including a myofilament component, may contribute to the greater slowing of relaxation in RVCMs. Accordingly, alterations in myofilament Ca^2+^ buffer properties may act not merely as secondary modifiers but as primary physiological determinants of contractile kinetics.

From this perspective, the effects of nNOS inhibition on Ca^2+^ transients and myofilament shortening in both LVCMs and RVCMs can be interpreted primarily in terms of a change in apparent endogenous Ca^2+^ buffering. Following SMTC treatment, the decay of the Ca^2+^ transient was modestly but significantly slowed in both cell types (Fig. 3), as reflected by a statistically significant prolongation of the time to 90% relaxation. The reduction in the apparent dissociation constant ($K_{d,S}$) of the endogenous Ca^2+^ binding species in both LVCMs and RVCMs suggests that an increase in Ca^2+^ buffer affinity contributes, at least in part, to the observed slowing of Ca^2+^ decay. Previous studies have reported variable effects of nNOS inhibition on SERCA function (Wang et al., 2008; Carnicer et al., 2013; Vielma et al., 2016). Thus, it is plausible that altered apparent endogenous Ca^2+^ buffering, potentially including a myofilament component, and a modest decrease in SERCA-mediated Ca^2+^ reuptake both contribute to the slowing of Ca^2+^ transient decay observed under nNOS inhibition.

Notably, the magnitude of the change in Ca^2+^ transient decay observed in the present study was smaller than that reported in several previous studies (Sears et al., 2003; Wang et al., 2008). This likely reflects our use of a relatively low concentration of SMTC (100 nM), chosen deliberately in light of reports indicating off-target effects of SMTC on other NOS isoforms at higher concentrations (Narayanan and Griffith, 1994; Awasthi et al., 2026). Importantly, even at this low concentration, clear and reproducible signaling effects were detected (Fig. 7).

### Interpreting the Ca^2+^–SL relationship

Our quantitative analysis revealed a specific RVCM phenotype following nNOS inhibition: (1) a modest increase in Ca^2+^ transient peak, but (2) a larger increase in sarcomere shortening and (3) a significant reduction in diastolic SL. When integrated, these findings indicate a shift in the Ca^2+^-SL relationship toward higher Ca^2+^ sensitivity.

It is important to acknowledge that our real-time [Ca^2+^]_c_–SL loop analysis differs from conventional steady-state pCa–force measurements. The dynamic nature of the contraction cycle involves hysteresis, and we deliberately excluded the systolic phase, during which nonlinear and highly dynamic interactions dominate. Thus, our analysis focused on the relaxation phase and diastolic levels where conditions approach quasi-equilibrium. Within these limitations, the data clearly demonstrate that in RVCMs, a greater contractile response is elicited at equivalent or even lower [Ca^2+^]_c_ following nNOS inhibition. This pattern is consistent with an increase in calcium sensitivity in the classical sense. However, our buffering data suggest this should be reinterpreted: the RV phenotype is driven by a change in the efficiency of Ca^2+^-contraction coupling, mediated by altered buffering capacity (increased bound fraction), rather than simply by the magnitude of the Ca^2+^ transient. This highlights the value of simultaneously measuring sensitivity and buffering to provide a unified interpretation of cardiac mechanics. The reduction in diastolic SL without a substantial increase in diastolic free Ca^2+^ also suggests persistent mechanical activation involving altered thin-filament regulation or crossbridge kinetics. The decrease in apparent $K_{d,S}$ is directionally consistent with greater endogenous Ca^2+^ binding at a given free Ca^2+^ concentration and may contribute to this phenotype. However, the present measurements do not establish whether the magnitude of the buffering shift is quantitatively sufficient to account for the observed reduction in diastolic SL, because endogenous Ca^2+^ binding could not be solely related to troponin occupancy.

The buffering analysis provides complementary but distinct information. The reduction in apparent *K*_d,S_ indicates altered apparent Ca^2+^-binding behavior of the combined endogenous buffering system, but it does not directly demonstrate an increase in the biochemical Ca^2+^ affinity of cTnC. Together with the reduction in p-cTnI, the changes in the Ca^2+^-SL relationship and apparent endogenous buffering properties are consistent with altered myofilament Ca^2+^ regulation in the SMTC-treated RVCMs. Thus, the Ca^2+^–SL relationship provides a functional index of Ca^2+^ sensitivity, whereas the added-buffer analysis describes the apparent behavior of the endogenous buffering system. It has to be noted that neither measurement directly determines the biochemical Ca^2+^ affinity of isolated cTnC.

### Physiological implication of the nNOSβ-regulated Ca^2+^ sensitivity in RVCMs

The RV is structurally more compliant and operates under lower afterload conditions than the LV. The higher basal nNOSβ activity and consequent lower Ca^2+^ sensitivity (and lower buffering affinity) in RVCMs may serve a physiological role to preserve lusitropy and limit energetic cost under basal conditions. Higher myofilament-associated nNOSβ activity in RVCMs may provide a cellular mechanism that favors relaxation under basal conditions. Such regulation could contribute to RV diastolic function with markedly lower afterload than LV, although its significance in the mechanically loaded intact ventricle remains to be determined.

Alterations in this pathway may have implications for pathophysiology. In our study, nNOS inhibition produced cellular features that may be relevant to pathological RV remodeling, including reduced diastolic SL and impaired relaxation. In conditions like pulmonary hypertension or RV hypertrophy, if nNOS activity or cGMP-PKG signaling is impaired, the resulting loss of TnI phosphorylation could lead to “hypersensitivity” to calcium, contributing to diastolic dysfunction and energetic inefficiency. Whether disruption of this pathway contributes to RV dysfunction during pressure overload, and whether its restoration improves RV performance, should be tested in intact-heart and disease models.

### Limitations and conclusion

While this study provides novel insights into the compartmentalized role of nNOSβ in RVCMs, certain limitations warrant mention. Firstly, our experiments were conducted under basal conditions; the dynamic regulation of this pathway during adrenergic stimulation, exercise, or pathological stress remains to be elucidated. Secondly, while SMTC is widely used as a selective nNOS inhibitor, potential off-target effects or incomplete specificity cannot be entirely excluded. Thirdly, the SL measured in this study represents unloaded isolated cardiomyocytes and should not be considered equivalent to the end-diastolic SL of the mechanically loaded myocardium.

With regard to the diastolic SL, Bub et al. (2010) reported a modal SL of 1.95 µm in cardioplegically arrested, Langendorff-perfused rat hearts, compared with 1.71 µm in acutely isolated ventricular cardiomyocytes. However, this intact-heart value was obtained during cardioplegic arrest and was influenced by progressive edema, and therefore it should not be regarded as a direct measurement of physiological end-diastolic SL. Studies of intact rat LV myocardium have also shown that diastolic SL varies with ventricular filling pressure, transmural location, and residual wall stress (Grimm et al., 1983; Rodriguez et al., 1993). Moreover, direct side-by-side measurements of physiological end-diastolic SL in the normal rat RV and LV remain limited. The absolute SL and magnitude of the responses observed here therefore cannot be directly extrapolated to the mechanically loaded myocardium.

Despite the limitations mentioned above, isolated cardiomyocytes provide a useful model for comparing cellular phenotypes of LVCMs and RVCMs under identical experimental conditions. Previous studies have demonstrated that chamber-specific differences in action-potential configuration, Ca^2+^ handling, contraction, troponin expression, and intracellular Ca^2+^ buffering are retained after isolation (Kondo et al., 2006; Jeon et al., 2022). In the present study, cells were collected separately from the free walls of the RV and LV, excluding the interventricular septum, thereby allowing direct comparison of chamber-specific cellular properties. However, isolated cells do not reproduce physiological preload and afterload, the extracellular matrix, myocardial architecture, ventricular interaction, or neurohumoral regulation. Thus, our findings identify a chamber-specific cardiomyocyte phenotype that remains detectable after isolation, although its quantitative contribution to intact ventricular function remains to be established. Importantly, its detection after isolation does not establish that the phenotype is independent of prior *in vivo* mechanical loading or neurohumoral signaling.

In conclusion, our findings reveal a more pronounced role for myofilament-associated nNOSβ in the RV. By acting locally to phosphorylate cTnI Ser^23/24^, nNOSβ acts as a crucial “brake” on myofilament Ca^2+^ sensitivity, thereby facilitating relaxation and maintaining diastolic compliance. This chamber-specific mechanism emphasizes that cardiac NO signaling is not uniform but is finely tuned by subcellular localization to meet the distinct hemodynamic demands of the RV.

## Supplementary Material

Review History

SourceData F1 is the source file for Fig. 1.

SourceData F7 is the source file for Fig. 7.

SourceData F8 is the source file for Fig. 8.

SourceData FS1 is the source file for Fig. S1.

## Data Availability

The numerical data underlying Figs. 1–8 and S1–S4, including individual animal- and cell-level values, are available from the corresponding author upon request.
