# Supplementary material for: Chamber-specific regulation of myofilament Ca2+ sensitivity by nNOSβ via troponin I phosphorylation: Predominance in the right ventricle
Source: J Gen Physiol. 2026 Oct 5;158(6):e202613982. doi: 10.1085/jgp.202613982 (PMC13637471; doi:10.1085/jgp.202613982)
Supplement: Supplementary file 1 [file jgp_202613982_review_history.pdf]

Chamber-Specific Regulation of Myofilament Ca2+ Sensitivity by nNOSβ via Troponin I Phosphorylation: Predominance in the Right Ventricle

Jae Won KWON, Hyerim Park, Ryeon Heo, Jihyun Jang, Jintae Kim, Seung Beom Oh, Yin Hua Zhang, Sung Joon Kim, and Young Keul Jeon

Corresponding Author(s): Young Keul Jeon, Seoul National University and Sung Joon Kim, Seoul National University

Review Timeline:

Submission Date:	February 15, 2026
Editorial Decision:	March 24, 2026
Revision Received:	August 4, 2026
Editorial Decision:	August 31, 2026
Revision Received:	September 2, 2026

Editor: David Eisner

Transaction Report:

(Note: With the exception of the correction of typographical or spelling errors that could be a source of ambiguity, letters and reports are not edited. The original formatting of letters and referee reports may not be reflected in this compilation.)

DOI: https://doi.org/10.1085/jgp.202613982

March 24, 2026

Dr. Young Keul Jeon
Seoul National University
Physiology
Daehak-ro 103
Seoul 03080
Korea, Republic of (South Korea)

Re: 202613982

Dear Dr. Jeon,

Thank you for submitting your manuscript, entitled "Chamber-Specific Regulation of Myofilament Ca2+ Sensitivity by nNOSβ via Troponin I Phosphorylation: Predominance in the Right Ventricle" to JGP. Your manuscript has now been seen by 3 reviewers, whose comments are appended below. You will see that the reviewers were enthusiastic about the study and its potential impact but raised some concerns that should nevertheless be addressed prior to further consideration of the manuscript at JGP. In particular, please note the comments of reviewer #1 concerning the upstream mechanism and physiological relevance to LV vs RV function as well as the comments on statistics made by the other reviewers.

We hope that you will be able to submit a revised manuscript that addresses all the comments of the reviewers, which we believe will pose no problems, and which may be re-reviewed. In addition, please do not hesitate to contact me (via the editorial office) if you feel that a discussion of the reviewers' and editors' comments would be helpful.

Please submit your revised manuscript via the link below, along with a point-by-point letter that details your response to the reviewers' and editor's summary, as well as a copy of the text with alterations highlighted (boldfaced or underlined). If the article is eventually accepted, it would include a 'revised date' as well as submitted and accepted dates. If we do not receive the revised manuscript within one year, we will regard the article as having been withdrawn. We would be willing to receive a revision of the manuscript at a later time, but the manuscript will then be treated as a new submission, with a new manuscript number.

Please pay particular attention to recent changes to our instructions to authors in the following sections: Data presentation, Blinding and randomization and Statistical analysis, under Materials and Methods, as shown here: https://rupress.org/jgp/pages/submission-guidelines#prepare. Re-review will be contingent on inclusion of the required information (including for data added during revision) and demonstration of the experimental reproducibility of the results. Also, To improve the reproducibility of published content, we have partnered with SciScore. Authors are prompted in eJP to copy and paste the Materials and Methods section of their manuscript for a SciScore assessment when submitting their revised manuscript. Authors are encouraged (not required) to further revise their Materials and Methods if the SciScore is below 4. More information can be found here: https://rupress.org/jgp/pages/submission-guidelines#sciscore.

Please note, JGP now requires authors to submit Source Data used to generate figures containing gels and Western blots with all revised manuscripts (when applicable). This Source Data consists of fully uncropped and unprocessed images for each gel/blot displayed in the main and supplemental figures. If your paper includes cropped gel and/or blot images, please be sure to provide one Source Data file for each figure that contains gels and/or blots along with your revised manuscript files. File names for Source Data figures should be alphanumeric without any spaces or special characters (i.e., SourceDataF#, where F# refers to the associated main figure number or SourceDataFS# for those associated with Supplementary figures). The lanes of the gels/blots should be labeled as they are in the associated figure, the place where cropping was applied should be marked (with a box), and molecular weight/size standards should be labeled wherever possible.
Source Data files will be made available to reviewers during evaluation of revised manuscripts and, if your paper is eventually published in JGP, the files will be directly linked to specific figures in the published article.

Source Data Figures should be provided as individual PDF files (one file per figure). Authors should endeavor to retain a minimum resolution of 300 dpi or pixels per inch. Please review our instructions for export from Photoshop, Illustrator, and PowerPoint here: https://rupress.org/jgp/pages/submission-guidelines#revised

Whilst you are revising your manuscript, we ask that you consider whether you have any artwork that might be suitable for the cover of JGP. Microscopy images are particularly good for cover artwork, but other types of image can be very effective, so we encourage you to be creative. Please don't restrict yourself to images from the paper; an image that is relevant to the work described would be just as suitable. Images should be a minimum resolution of 300 dpi. To see recent examples, visit the following page and click on 'Show covers? Yes': https://jgp.rupress.org/content/by/year)

Thank you for submitting your interesting research to JGP.

Please submit your revised manuscript, and any associated files, via this link:
Link Not Available

Sincerely,

David Eisner, D. Phil
On behalf of Journal of General Physiology

Journal of General Physiology's mission is to publish mechanistic and quantitative molecular and cellular physiology of the highest quality; to provide a best-in-class author experience; and to nurture future generations of independent researchers.

Reviewer #1 (Comments to the Authors):

This is a careful study of E-C coupling in isolated cardiac cells from rat heart focussing on the comparison of E-C coupling processes between cardiac cells from the right and left ventricle.
The electrophysiology and intracellular calcium measurements are technically of very high quality and the statistical methods of analysis appears appropriate.
The data demonstrates a differential with respect to nNOSbeta phosphorylation state, TnI phosphorylation and functional parameters such as cell shortening parameters and an overall buffer power of the cytosol (indirectly assessing myofilament Ca sensivity).
In my opinion, this study has only a few technical issues to consider in a revision, but the authors should also consider revising the Introduction and Discussion to point to the underlying physiology of the two chambers that points to a different E-C coupling/haemodynamic status of the RV and LV, i.e. the authors should do more to put their data into a physiological context. This includes the upstream mechanisms for the different nNOS status across the two ventricles.
Criticisms:
1. While the authors show a difference in the local (myofilament) phosphorylation status of nNOS, there is no discussion of the upstream systems that are responsible. As they know, nNOS phosphorylation is determined by several factors. This response should be integrated with a better description of prior work on the RV/LV haemodynalic differences. Included in this is the important information on the end diastolic sarcomere length in-situ in the LV and RV. How does that compare with the isolated cell values? What evidence is there that single cells are a good model of the myocardium in the respective chambers.
2. The calcium buffer analysis is very well done, but one puzzling feature is that the pipette solution has 0.1mM EGTA and yet there appears to be no consideration of the contribution of EGTA to the total intracellular buffer signal (after Fura has been subtracted). To what extent does EGTA contribute to the Stotal? (100uM?) - please explain and if it is assumed to be negligible, what experimental evidence is there for this?
3. The buffer analysis data jumps straight into the derived buffer power plots. I understand that this is because the method is described in detail in 2022 paper. But I would suggest that the authors include a supplementary figure that shows an example of the experimental traces that go into these measurements.

Reviewer #2 (Comments to the Authors):

Thank you for the opportunity to review this manuscript. I found the study interesting and enjoyed reading the work. The experiments are carefully performed and the data are generally of high technical quality. The integration of sarcomere shortening, Ca^{2+} transient measurements, electrophysiology, quantitative buffering analysis, and biochemical assessment provides a coherent and internally consistent dataset. The manuscript builds logically on the group's prior work examining interventricular differences in myofilament regulation and extends this into nitric oxide-dependent modulation of chamber-specific function.
The quantitative assessment of endogenous Ca^{2+} buffering is a particular strength of the manuscript. The observation that nNOS inhibition produces a disproportionately large increase in contractility in right ventricular cardiomyocytes, despite relatively modest changes in Ca^{2+} transient amplitude and ICaL, is physiologically compelling. The integration of Ca^{2+}sarcomere loop analysis with buffering parameters strengthens the argument that altered myofilament interaction with Ca^{2+} contributes to the chamber-specific phenotype.
I do, however, have several comments regarding interpretation, statistical reporting, and mechanistic framing that I believe would strengthen the manuscript.

Major

1. Interpretation of buffering parameters (κS and Kd,S)

The buffering analysis is central to the mechanistic interpretation and requires careful phrasing. κS and Kd,S derived from the added-buffer approach reflect the apparent behaviour of the endogenous buffering system under intracellular conditions. They do not directly measure the biochemical affinity of troponin C in isolation (reasonably so.) In several sections of the Discussion, reductions in Kd,S are described in a manner that could be interpreted as demonstrating altered troponin C affinity. While this is a reasonable hypothesis, it remains inferential. The manuscript would benefit from clearer distinction between apparent buffering parameters, functional Ca^{2+} sensitivity derived from Ca^{2+}-sarcomere loop analysis, and direct biochemical affinity of regulatory proteins. More precise framing would improve mechanistic clarity without weakening the overall conclusions.

2. Role of Mg^{2+} and intracellular assumptions

Because EF-hand proteins, including troponin C, bind Mg^{2+} at structural sites, Mg^{2+} occupancy can influence apparent Ca^{2+} affinity under intracellular conditions. It would therefore be helpful if the authors briefly clarified the intracellular Mg^{2+} assumptions relevant to interpretation of Kd,S, and whether Mg^{2+}-dependent effects were considered in analysing or interpreting the apparent affinity shifts. Even a short clarification would strengthen the underpinning of the buffering data, particularly when relatively modest shifts in apparent affinity are interpreted as mechanistically meaningful.

3. Linking buffering changes to mechanical phenotype

The reduction in diastolic sarcomere length in right ventricular cardiomyocytes following nNOS inhibition is an interesting finding, particularly in the absence of substantial changes in diastolic free Ca^{2+} levels. This suggests altered thin filament regulation or cross-bridge kinetics rather than altered free Ca^{2+} availability. The manuscript would be strengthened by briefly addressing whether the measured shift in buffering parameters is quantitatively sufficient to account for the observed mechanical change.

4. Quantitative comparison of ICaL, Ca^{2+} transients, and shortening

The argument that enhanced contractility in RVCMs cannot be explained solely by changes in Ca^{2+} influx is plausible. While the individual changes in ICa,L, Ca^{2+} transient amplitude, and sarcomere shortening are reported, the argument would be strengthened by explicitly comparing the relative magnitude of these changes within the Results. Presenting the proportional differences more directly would make the logic underlying the conclusion clearer.

5. Myofilament fractionation and localisation

The enrichment of phosphorylated nNOSβ in the myofilament fraction is an important component of the proposed mechanism. The fractionation protocol and normalisation strategy are described. Given that subcellular localisation is central to the chamber-specific interpretation, if possible, it would be helpful to either briefly reference prior validation of this fractionation method or include a concise statement confirming fraction purity.

6. Statistical reporting

The overall statistical approach appears appropriate, including the use of nested analysis to account for cells grouped within animals. However, greater transparency would strengthen the manuscript. Exact p values should be reported where feasible rather than relying solely on threshold-based asterisk notation. The numbers of animals (N) and cells (n) should be consistently stated in figure panels, and the specific statistical test (e.g two tail vs one tail t test) used for each comparison should be clearly indicated in the corresponding figure legend. If normality testing was performed, this should also be stated.

Minor

Abbreviations should be defined at first mention in the main text, even if introduced in the abstract. In several places, the Discussion slightly overstates causality where association has been demonstrated; rephrasing to reflect this would improve scientific precision. Finally, the broader physiological implications, particularly with respect to potential relevance to right ventricular function under stress or disease conditions, could be framed slightly more clearly while remaining within the limits of the presented data.

Overall

This is a carefully performed and physiologically interesting study that advances understanding of chamber-specific regulation of myofilament Ca^{2+} handling. The integration of functional measurements with quantitative buffering analysis is a strength. With refinement of statistical reporting and more precise mechanistic framing, the manuscript would be further strengthened.

Reviewer #3 (Comments to the Authors):

Kwon and colleagues, build on the findings of their previous work (2022) to investigate chamber-specific differences in ventricular cardiomyocyte responses to the excitation-contraction coupling regulator neuronal nitric oxide synthase (nNOS). The authors report enhanced sarcomere shortening but impaired relaxation in both left and right ventricular cardiomyocytes (LVCMs and RVCMs, respectively) following pharmacological blockade of nNOS, with these alterations being more pronounced in RVCMs. In contrast, However, calcium handling changes particularly ICaL and Ca2+ transient, were reported to be more pronounced in the LVCM compared to the RCVM, highlighting a dissociation between the Ca2+ signalling and the corresponding mechanical output in RCVMs. The authors further propose the increased myofilament Ca2+ senisitvity/buffering in RCVMs, driven by enhanced nNOS mediated phosphorylation of cTnI, as the differing factor.mediating these chamber specific differences.
The manuscript is well written and the experiments appear carefully designed. However, there is a major statistical concern that is applied broadly throughout the manuscript which needs to be addressed to ensure that the findings are properly interpreted and conveyed. Please find my specific comments below

1. The central theme of the manuscript is the comparison of the differential response of excitation-contraction coupling players to nNOS inhibition in LVCMs and RVCMs (i.e., between chambers). However, the statistical analysis is largely limited to within chamber comparisons (baseline vs. inhibitor-treated), which is insufficient to support the chamber-specific conclusions drawn throught the manuscript. While the authors apply appropriate statistics within groups (control vs. treatment), comparisons across chambers (RVCMs vs. LVCMs) are interpreted primarily on visual comparison. This approach is not adequate to substantiate claims of differential chamber specific effects. For example, in line 350 (page 15), the authors state that the SMTC-induced increase in mean ΔSL is significantly greater in RVCMs than in LVCMs; however, no statistical analysis supporting this comparison is provided. Similarly, in line 356 (page 15), the authors suggest that fractional shortening is markedly higher in RVCMs than in LVCMs and conclude that there is a preferential inotropic response, yet it is unclear on what statistical basis this conclusion is made. As currently analysed, it is not possible to determine whether the observed differences in effect size between chambers are statistically significant or simply reflect biological heterogeneity. A direct statistical comparison between chambers is therefore required. As such, statistical analysis that accounts for both treatment and chamber interaction (e.g two-way ANOVA or mixed-effects model) should be applied. Without this, the primary conclusions of the manuscript rely on informal visual comparisons between two separately tested groups.

2. In line 350 (page 15) the the bracketed ΔSL values i.e 31.2 um and 21.6um are wrong and should be corrected.

3. In line 381 (page 16) the statement "in contrast, these effects were modest and did not reach statistical significance in RCVMs (Fig 3D, E)" doesn't appear to be so, as the figures say otherwise

Editor's comments

1. Please note the comments of reviewer #1 concerning the upstream mechanism and physiological relevance to LV vs RV function as well as the comments on statistics made by the other reviewers.

   Response: We thank the editor for highlighting these important points. We made particular efforts to address Reviewer #1's comments regarding the upstream mechanisms and physiological relevance, as well as the statistical concerns raised by the other reviewers.
   Briefly, we expanded the Introduction and Discussion to describe the different mechanical and hemodynamic environments of the RV and LV and to discuss possible upstream pathways regulating myofilament-associated nNOSβ phosphorylation, including mechanosensitive AMPK signaling, β3-adrenergic–Akt signaling, and chamber-dependent adrenergic signal compartmentation.
   We also clarified the relationship between sarcomere length in isolated cardiomyocytes and intact myocardium, the limitations of the isolated-cell model, and the potential relevance of this pathway to RV stress and disease without overextending the conclusions.
   Finally, we added direct chamber-by-treatment analyses using two-way repeated-measures ANOVA on animal-level means, reported the interaction statistics, and revised the Results and Discussion wherever the between-chamber difference was not statistically significant.

2. Please pay particular attention to recent changes to our instructions to authors in the following sections: Data presentation, Blinding and randomization and Statistical analysis, under Materials and Methods, as shown here: https://rupress.org/jgp/pages/submission-guidelines#prepare. Re-review will be contingent on inclusion of the required information (including for data added during revision) and demonstration of the experimental reproducibility of the results.

   Response: We carefully revised the Materials and Methods and figure legends according to the updated JGP requirements for data presentation, blinding and randomization, and statistical analysis. We clarified the definitions of biological and cellular replicates, displayed individual data points and animal-level means where appropriate, and provided *N* and *n* consistently.

3. Please note, JGP now requires authors to submit Source Data used to generate figures containing gels and Western blots with all revised manuscripts (when applicable). This Source Data consists of fully uncropped and unprocessed images for each gel/blot displayed in the main and supplemental figures. If your paper includes cropped gel and/or blot images, please be sure to provide one Source Data file for each figure that contains gels and/or blots along with your revised manuscript files. File names for Source Data figures should be alphanumeric without any spaces or special characters (i.e., SourceDataF#, where F# refers to the associated main figure number or SourceDataFS# for those associated with Supplementary figures). The lanes of the gels/blots should be labeled as they are in

the associated figure, the place where cropping was applied should be marked (with a box), and molecular weight/size standards should be labeled wherever possible.

Source Data files will be made available to reviewers during evaluation of revised manuscripts and, if your paper is eventually published in JGP, the files will be directly linked to specific figures in the published article. Source Data Figures should be provided as individual PDF files (one file per figure). Authors should endeavor to retain a minimum resolution of 300 dpi or pixels per inch. Please review our instructions for export from Photoshop, Illustrator, and PowerPoint here: https://rupress.org/jgp/pages/submission-guidelines#revised

Response: Thank you for the detailed instructions. As requested, we prepared Source Data files containing the uncropped blot images for Figures 1, 7, and 8, as well as Supplementary Figure 1. During preparation of the Source Data files, we found that some of the relatively recent blot images had been saved only as small, low-resolution files. We therefore replaced these representative blot images with higher-resolution photographs containing the corresponding full gels or blots. Consequently, some blot images shown in the main figures were replaced. However, the underlying experimental data used for quantification and the corresponding bar graphs were not changed.

Reviewer #1's comments

1. While the authors show a difference in the local (myofilament) phosphorylation status of nNOS, there is no discussion of the upstream systems that are responsible. As they know, nNOS phosphorylation is determined by several factors. This response should be integrated with a better description of prior work on the RV/LV haemodynalic differences. Included in this is the important information on the end diastolic sarcomere length in-situ in the LV and RV. How does that compare with the isolated cell values? What evidence is there that single cells are a good model of the myocardium in the respective chambers.

   Response: We thank the reviewer for this important and thoughtful comment. We agree that the original manuscript did not sufficiently discuss the possible upstream mechanisms responsible for the chamber-specific phosphorylation of myofilament-associated nNOSβ. We also agree that the physiological differences between the RV and LV, as well as the limitations of the isolated-cell preparation, required a clearer discussion.
   First, we added a description of the different mechanical and hemodynamic environments of the RV and LV in the fourth paragraph of the Introduction, beginning with "Despite these extensive studies…". This revision includes the different pressure-volume relationships and contraction patterns of the two ventricles.
   Second, we added a discussion of possible upstream pathways regulating nNOS phosphorylation in the third paragraph of the Discussion subsection, "Compartmentalized activation of nNOSβ underlies chamber-specific regulation of E-C coupling." We now discuss mechanosensitive AMPK signaling, β_3-adrenergic–Akt signaling, and chamber-dependent adrenergic signal compartmentation as possible mechanisms. We also clearly state that these pathways were not directly examined in the present study and that the mechanism responsible for the higher phosphorylation of myofilament-associated nNOSβ in RVCMs remains unresolved.
   Third, we added the comparison between sarcomere length in isolated cardiomyocytes and intact myocardial preparations in the second paragraph of "Limitations and Conclusion." We now clarify that the sarcomere lengths measured in our unloaded isolated cells are shorter than those reported in intact rat myocardial preparations and should not be considered equivalent to physiological end-diastolic sarcomere length. We also note that direct side-by-side measurements of physiological end-diastolic sarcomere length in the normal rat RV and LV remain limited. In the same paragraph, we added the rationale and limitations of using isolated cardiomyocytes. Previous studies support that isolated RV and LV cardiomyocytes retain intrinsic chamber-specific differences. However, we now clearly acknowledge that this model does not reproduce physiological loading, myocardial architecture, intercellular interactions, or neurohumoral regulation.
   Finally, we revised the "Physiological Implication of the nNOSβ-regulated Ca^{2+} Sensitivity in RVCMs" subsection and the concluding paragraph to avoid direct extrapolation from isolated

cardiomyocytes to whole-ventricle function. We now describe our findings as an intrinsic chamber-specific cellular mechanism whose physiological contribution in the mechanically loaded intact heart remains to be established.

2. The calcium buffer analysis is very well done, but one puzzling feature is that the pipette solution has 0.1mM EGTA and yet there appears to be no consideration of the contribution of EGTA to the total intracellular buffer signal (after Fura has been subtracted). To what extent does EGTA contribute to the Stotal? (100uM?) - please explain and if it is assumed to be negligible, what experimental evidence is there for this?

   Response: Thank you for this important comment. We agree that the presence of EGTA in the pipette solution could potentially contribute to the total intracellular buffering signal (S_{total}) and therefore requires clarification. We realize that our description of the “Cs-based pipette solution” may have caused confusion. In this study, we used two different Cs-based pipette solutions: (1) a Cs-pipette and (2) an $I_{Ca,L}$-pipette. Importantly, the calcium buffering experiments were performed using the Cs-pipette solution. To avoid any contribution from exogenous buffers such as EGTA, the Cs-pipette solution used for calcium buffer measurements did not contain EGTA. Instead, Fura-2 was intentionally used as the sole exogenous calcium buffer in these experiments. Therefore, EGTA does not contribute to S_{total} in our calcium buffering analysis. Because this is a critical methodological point, we have now clarified this explicitly in the revised manuscript (Methods – Calcium imaging technique for measuring Ca^{2+} binding ratio, first paragraph).

3. The buffer analysis data jumps straight into the derived buffer power plots. I understand that this is because the method is described in detail in 2022 paper. But I would suggest that the authors include a supplementary figure that shows an example of the experimental traces that go into these measurements.

   Response: We agree that the original figure presented the derived buffering parameters without showing the underlying experimental recording. We have therefore added a new supplementary figure showing one representative raw recording used for the buffering analysis. To avoid an overly complex and repetitive figure, one representative example is presented in the new Figure S4.

Reviewer #2's comments

1. Interpretation of buffering parameters (κS and Kd,S)

   The buffering analysis is central to the mechanistic interpretation and requires careful phrasing. κS and Kd,S derived from the added-buffer approach reflect the apparent behaviour of the endogenous buffering system under intracellular conditions. They do not directly measure the biochemical affinity of troponin C in isolation (reasonably so.) In several sections of the Discussion, reductions in Kd,S are described in a manner that could be interpreted as demonstrating altered troponin C affinity. While this is a reasonable hypothesis, it remains inferential. The manuscript would benefit from clearer distinction between apparent buffering parameters, functional Ca^{2+} sensitivity derived from Ca^{2+}-sarcomere loop analysis, and direct biochemical affinity of regulatory proteins. More precise framing would improve mechanistic clarity without weakening the overall conclusions.

   Response: We thank the reviewer for this careful and important comment. We agree that the original manuscript did not sufficiently distinguish among apparent endogenous Ca^{2+} buffering parameters, functional myofilament Ca^{2+} sensitivity, and the direct biochemical affinity of individual Ca^{2+}-binding proteins.

   In response, we reviewed the terminology and interpretation throughout the manuscript and revised several sections. We now consistently describe κ_S and $K_{d,S}$ as parameters representing the apparent behavior of the combined endogenous Ca^{2+} buffering system under intracellular conditions. We also clearly state that these parameters do not directly measure the biochemical Ca^{2+} affinity of an individual protein such as cTnC.

   We separately define the Ca^{2+}-sarcomere length relationship as a functional index of myofilament Ca^{2+} sensitivity. This analysis reflects the relationship between intracellular Ca^{2+} and the mechanical response in intact cardiomyocytes, but it is not equivalent to a direct biochemical binding assay or a conventional steady-state pCa-force measurement.

   We also revised the interpretation linking the buffering results to the troponin complex. Because cTnC is a major endogenous Ca^{2+} buffer and cTnI phosphorylation can influence Ca^{2+} binding to cTnC, we believe that altered troponin regulation remains a reasonable mechanistic interpretation. However, we now describe this as an inference supported by the parallel changes in apparent $K_{d,S}$, functional Ca^{2+} sensitivity, and cTnI phosphorylation, rather than as a direct measurement of cTnC affinity.

   We retained the central interpretation that altered endogenous Ca^{2+} buffering contributes importantly to the RVCM phenotype, because this interpretation is supported by several independent observations in our study. However, we revised the wording to make clear which findings were directly measured and which parts represent an integrated mechanistic interpretation.

   The major revisions are summarized below:

   Abstract: We replaced the general expression "myofilament Ca^{2+} binding properties" with "endogenous Ca^{2+} buffering properties."

Introduction: We now explicitly state that myofilament Ca^{2+} sensitivity and Ca^{2+} buffering are related but distinct properties. We also reframed the study question in terms of functional myofilament Ca^{2+} sensitivity and endogenous Ca^{2+} buffering rather than direct cTnC affinity.

Materials and Methods, "Calcium imaging technique for measuring Ca^{2+} binding ratio": We added a clear definition that κ_S and $K_{d,S}$ describe the apparent behavior of the combined endogenous buffering system and do not directly measure the biochemical affinity of cTnC or another individual buffer protein.

Results, "Quantification of endogenous Ca^{2+} buffering reveals chamber-specific modulation by nNOS": We revised the terminology to distinguish endogenous cytosolic buffering from direct myofilament or cTnC binding measurements. We also added a statement that the similarity between $K_{d,S}$ and reported cTnC values should not be interpreted as evidence that $K_{d,S}$ is a direct measurement of cTnC affinity.

Results, "nNOS inhibition preferentially reduces TnI phosphorylation in RVCMs": We revised the conclusion to state that the greater reduction in cTnI phosphorylation provides a potential molecular basis for the changes in functional Ca^{2+} sensitivity and apparent endogenous buffering, rather than directly proving an increase in cTnC affinity.

Discussion, "Myofilament Ca^{2+} buffering as a determinant of apparent Ca^{2+} sensitivity": We revised several statements to describe the added-buffer analysis as an estimate of apparent endogenous buffering properties. The possible contribution of altered Ca^{2+} binding within the troponin complex is now presented as a mechanistic interpretation supported by, but not directly isolated in, the present experiments.

Discussion, "Interpreting the Ca^{2+}-SL Relationship": We added a new paragraph clearly distinguishing the three concepts. The Ca^{2+}-SL relationship is described as a functional index of Ca^{2+} sensitivity, the added-buffer analysis describes the apparent behavior of the endogenous buffering system, and neither measurement directly determines the biochemical Ca^{2+} affinity of isolated cTnC.

We appreciate the reviewer's suggestion, which helped us make the mechanistic interpretation and terminology more precise.

2. Role of Mg^{2+} and intracellular assumptions

   Because EF-hand proteins, including troponin C, bind Mg^{2+} at structural sites, Mg^{2+} occupancy can influence apparent Ca^{2+} affinity under intracellular conditions. It would therefore be helpful if the authors briefly clarified the intracellular Mg^{2+} assumptions relevant to interpretation of Kd,S, and whether Mg^{2+}-dependent effects were considered in analyzing or interpreting the apparent affinity shifts. Even a short clarification would strengthen the underpinning of the buffering data, particularly when relatively modest shifts in apparent affinity are interpreted as mechanistically meaningful.

Response: We thank the reviewer for this important comment. We agree that Mg^{2+} occupancy should be considered when interpreting the apparent endogenous buffering parameters, particularly because the C-terminal structural sites of cTnC can bind both Ca^{2+} and Mg^{2+}.

We have clarified the Mg^{2+} composition and this assumption in the Methods section and added a short discussion of this limitation in the subsection "Myofilament Ca^{2+} buffering as a determinant of apparent Ca^{2+} sensitivity." We also now emphasize that the absolute $K_{d,S}$ values are conditional on the intracellular ionic environment of the whole-cell recording and should not be interpreted as biochemical affinity constants for cTnC.

3. Linking buffering changes to mechanical phenotype

   The reduction in diastolic sarcomere length in right ventricular cardiomyocytes following nNOS inhibition is an interesting finding, particularly in the absence of substantial changes in diastolic free Ca^{2+} levels. This suggests altered thin filament regulation or cross-bridge kinetics rather than altered free Ca^{2+} availability. The manuscript would be strengthened by briefly addressing whether the measured shift in buffering parameters is quantitatively sufficient to account for the observed mechanical change.

   Response: We thank the reviewer for this important point. We agree that the reduction in diastolic SL in the absence of a substantial increase in diastolic free Ca^{2+} suggests altered thin-filament regulation or cross-bridge kinetics. The decrease in apparent $K_{d,S}$ is directionally consistent with greater endogenous Ca^{2+} binding at a given free Ca^{2+} concentration (basal level) and may therefore contribute to the reduced diastolic SL. However, we acknowledge that the present data do not allow us to determine whether this relationship is quantitatively sufficient to explain the observed mechanical change. The added-buffer analysis does not separately determine cTnC occupancy, and the buffering and sarcomere measurements were obtained under different experimental conditions. We have therefore added a statement in the Discussion clarifying that the change in buffering is consistent with, but has not been shown to be quantitatively sufficient to explain, the full mechanical phenotype.

4. Quantitative comparison of ICaL, Ca^{2+} transients, and shortening

   The argument that enhanced contractility in RVCMs cannot be explained solely by changes in Ca^{2+} influx is plausible. While the individual changes in $I_{Ca,L}$, Ca^{2+} transient amplitude, and sarcomere shortening are reported, the argument would be strengthened by explicitly comparing the relative magnitude of these changes within the Results. Presenting the proportional differences more directly would make the logic underlying the conclusion clearer.

Response: We thank the reviewer for this helpful suggestion. We have added a direct comparison of the proportional changes in peak $I_{Ca,L}$ Ca^{2+} transient peak amplitude, and sarcomere shortening in the Results subsection “Chamber-specific effects of nNOS inhibition on Ca^{2+} handling.”

5. Myofilament fractionation and localization
The enrichment of phosphorylated nNOSβ in the myofilament fraction is an important component of the proposed mechanism. The fractionation protocol and normalisation strategy are described. Given that subcellular localisation is central to the chamber-specific interpretation, if possible, it would be helpful to either briefly reference prior validation of this fractionation method or include a concise statement confirming fraction purity.

Response: We thank the reviewer for this helpful suggestion. We have added a brief clarification in the Materials and Methods subsection, **“**Isolation of membrane/cytosolic and myofilament-enriched fraction.” The myofilament-enriched fraction was prepared using the previously established protocol of Jang et al. (2015), in which fraction purity was validated by the absence of detectable GAPDH, a cytosolic marker, in the myofilament fraction, as shown in Figure 5A of that study.

6. Statistical reporting
The overall statistical approach appears appropriate, including the use of nested analysis to account for cells grouped within animals. However, greater transparency would strengthen the manuscript. Exact p values should be reported where feasible rather than relying solely on threshold-based asterisk notation. The numbers of animals (N) and cells (n) should be consistently stated in figure panels, and the specific statistical test (e.g two tail vs one tail t test) used for each comparison should be clearly indicated in the corresponding figure legend. If normality testing was performed, this should also be stated

Response: We revised the Statistical Analysis section to specify the use of two-tailed t-tests and two-tailed nested t-tests, with cells nested within animals where appropriate. Exact *P* values are now provided in the figures or figure legends wherever feasible, with only values below 0.0001 reported as $P < 0.0001$. We also added the numbers of animals and cells and specified the statistical test used for each comparison in the corresponding figure panels and legends.

7. Abbreviations should be defined at first mention in the main text, even if introduced in the abstract. In several places, the Discussion slightly overstates causality where association has been demonstrated; rephrasing to reflect this would improve scientific precision. Finally, the broader physiological implications, particularly with respect to potential relevance to right ventricular function

under stress or disease conditions, could be framed slightly more clearly while remaining within the limits of the presented data.

Response: We thank the reviewer for these detailed comments. Following the reviewer's suggestions, we defined the abbreviations that were missing at their first appearance in the main text, including PKA, PKG, cAMP, and SERCA2a. We also revised several statements in the Discussion that could imply direct causality, so that they now more accurately distinguish the observed associations from mechanistic interpretation. Finally, we clarified the possible relevance of this pathway to RV stress and disease conditions while clearly stating that these implications remain to be tested in intact-heart and disease models.

Reviewer #3's comments

1. The central theme of the manuscript is the comparison of the differential response of excitation-contraction coupling players to nNOS inhibition in LVCMs and RVCMs (i.e., between chambers). However, the statistical analysis is largely limited to within chamber comparisons (baseline vs. inhibitor-treated), which is insufficient to support the chamber-specific conclusions drawn throught the manuscript. While the authors apply appropriate statistics within groups (control vs. treatment), comparisons across chambers (RVCMs vs. LVCMs) are interpreted primarily on visual comparison. This approach is not adequate to substantiate claims of differential chamber specific effects. For example, in line 350 (page 15), the authors state that the SMTC-induced increase in mean ΔSL is significantly greater in RVCMs than in LVCMs; however, no statistical analysis supporting this comparison is provided. Similarly, in line 356 (page 15), the authors suggest that fractional shortening is markedly higher in RVCMs than in LVCMs and conclude that there is a preferential inotropic response, yet it is unclear on what statistical basis this conclusion is made. As currently analysed, it is not possible to determine whether the observed differences in effect size between chambers are statistically significant or simply reflect biological heterogeneity. A direct statistical comparison between chambers is therefore required. As such, statistical analysis that accounts for both treatment and chamber interaction (e.g two-way ANOVA or mixed-effects model) should be applied. Without this, the primary conclusions of the manuscript rely on informal visual comparisons between two separately tested groups.

   Response: We thank the reviewer for this important comment. We agree that separate within-chamber comparisons alone are insufficient to establish a chamber-specific response. We therefore retained the two-tailed nested t-tests for comparisons between control and SMTC-treated cells within each chamber, but added a direct statistical comparison of the treatment effects between chambers. For this analysis, cell-level measurements were averaged within each animal and experimental condition, and the resulting animal-level means were analyzed using two-way repeated-measures ANOVA with chamber and treatment as within-animal factors. The chamber-by-treatment interaction was used to determine whether the response to SMTC differed significantly between LVCMs and RVCMs.

   The Results and Statistical Analysis sections have been revised accordingly. Exact interaction statistics are now provided for the principal contractile and relaxation parameters. For example, significant chamber-by-treatment interactions were observed for ΔSL ($F_{1,9}$ = 24.13, p = 0.0008) and diastolic ($F_{1,9}$ = 6.15, p = 0.0349), supporting greater SMTC-induced changes in RVCMs. In contrast, although the prolongation of late relaxation was numerically greater in RVCMs, the chamber-by-treatment interaction was not significant ($F_{1,9}$ = 2.80, p = 0.1284); therefore, the corresponding interpretation was revised to avoid claiming a statistically significant chamber-specific difference. We similarly added direct interaction analyses to the sections describing Ca^{2+} transient amplitude and

decay kinetics, and revised all related statements according to whether the chamber-by-treatment interaction was significant. Thus, chamber-specific conclusions are now based on direct statistical comparisons rather than visual comparison of separately tested groups.

2. In line 350 (page 15) the bracketed ΔSL values i.e 31.2 um and 21.6um are wrong and should be corrected.

   Response: Thank you for pointing this out. This was an important oversight, and we have now corrected it in the revised manuscript.

3. In line 381 (page 16) the statement "in contrast, these effects were modest and did not reach statistical significance in RCVMs (Fig 3D, E)" doesn't appear to be so, as the figures say otherwise.

   Response: We thank the reviewer for pointing this out. The statement was based on values from an earlier version of the statistical analysis and was inadvertently retained after the data were reanalyzed using two-tailed nested t-tests. We have now corrected the Figure 3.

Dr. Young Keul Jeon
Seoul National University
Physiology
Daehak-ro 103
Seoul 03080
Korea, Republic of (South Korea)

Re: 202613982R1

Dear Dr. Jeon,

I am pleased to let you know that your manuscript, titled "Chamber-Specific Regulation of Myofilament Ca2+ Sensitivity by nNOSβ via Troponin I Phosphorylation: Predominance in the Right Ventricle" is scientifically acceptable for publication in Journal of General Physiology. Formal acceptance will follow when it is modified in accordance with the referees' remarks and our editorial policies. Please note the useful suggestion from reviewer #1.

Please note items that need attention are listed at the bottom of this email (under 'manuscript formatting checklist'). Please also be sure to include a letter addressing the reviewers' comments point-by-point (if applicable) and a copy of the text with alterations highlighted (boldfaced or underlined). Your manuscript should be a double-spaced MS Word file and include editable tables, if appropriate.

Lastly, JGP requires a data availability statement for all research article submissions. These statements will be published in the article directly above the Acknowledgments. The statement should address all data underlying the research presented in the manuscript. Please visit the JGP instructions for authors for guidelines and examples of statements at https://rupress.org/jgp/pages/editorial-policies#data-availability-statement.

Please submit your final files via this link:
Link Not Available

Thank you for choosing to publish your research in JGP and please feel free to contact me with any questions.

Sincerely,

David Eisner, D. Phil
On behalf of Journal of General Physiology

Journal of General Physiology's mission is to publish mechanistic and quantitative molecular and cellular physiology of the highest quality; to provide a best in class author experience; and to nurture future generations of independent researchers.

Manuscript formatting checklist:
- MS Word document of text needed (including editable tables)
- MS Word document of supplemental text needed, if applicable (including figure legends and editable tables)
- Brief Statement describing supplementary information needed, if applicable (in subsection at end of Materials & Methods)
- Please include a data availability statement preceding the Acknowledgments section. Please see https://rupress.org/jgp/pages/editorial-policies#data-availability-statement
- Figures created at sufficient resolution and in acceptable format (including supplemental if applicable). If working in Illustrator, we prefer .ai or .eps file format. If working in Photoshop please use 600dpi/1000dpi .tiff or .psd file format. Minimum resolution at estimated print size: Minimum resolution for all figures is 600 dpi. For figures that contain both photographs and line art or text, 600 dpi is highly recommended. Figures containing only black and white elements (line art, no color, and no gray) should be 1,000 dpi. Maximum figure size is 7 in wide x 9 in high (17.5 x 22.8 cm) at the correct resolution. https://jgp.rupress.org/fig-vid-guidelines
- Supplemental figures, if any, conforming to same guidelines as manuscript figures (noted above)
- If images resemble one from a prior publications, the author must seek permissions (to reproduce or adapt) from the original publisher. [You can resubmit your paper while waiting to hear back from the original publisher but please keep us updated]
- All authors must complete a disclosure form prior to acceptance. A link to complete the form has been sent to all coauthors. Please provide the editorial office with updated email addresses if necessary

---

Reviewer #1 (Comments to the Authors):

The authors have addressed the concerns expressed in the earlier review.
I have one minor further comment: I think it's important when discussing the upstream mechanisms of the altered nNOSβ phosphorylation, to mention mechanisms that may act acutely (mechanical load or require membrane-receptor mediated mechanisms) and those that would be retained chronically when the cardiomycytes are isolated and placed in standard media for a considerable period. The mechanisms that are relevant are ones that are not mediated by an acute mechanisms, that would be retained in vitro. This point needs to be considered in the revised ms..

Reviewer #2 (Comments to the Authors):

Thank you for your resubmission. I am happy you addressed my comments. Thank you for your contribution.

Reviewer #3 (Comments to the Authors):

The revised manuscript is responsive and has addressed my concerns and critiques. I am satisfied with the revisions and the additional analysis performed by the authors and, as such i have no further comments.

Editor's comments

1. I am pleased to let you know that your manuscript, titled "Chamber-Specific Regulation of Myofilament Ca^{2+} Sensitivity by nNOSβ via Troponin I Phosphorylation: Predominance in the Right Ventricle" is scientifically acceptable for publication in Journal of General Physiology. Formal acceptance will follow when it is modified in accordance with the referees' remarks and our editorial policies. Please note the useful suggestion from reviewer #1.

   Response: We sincerely thank the Editor for the positive evaluation of our manuscript and for the opportunity to revise it for publication in the Journal of General Physiology. We are also grateful to the Editor and reviewers for their careful evaluation and constructive comments throughout the review process, which have been invaluable in refining the manuscript and clarifying the interpretation and presentation of our findings.

   We carefully considered Reviewer #1's helpful suggestion and revised the Discussion accordingly.

   We have also reviewed the manuscript and accompanying files for compliance with the Journal's editorial policies. We hope that these revisions fully address the remaining comment.

2. Lastly, JGP requires a data availability statement for all research article submissions. These statements will be published in the article directly above the Acknowledgments. The statement should address all data underlying the research presented in the manuscript. Please visit the JGP instructions for authors for guidelines and examples of statements at https://rupress.org/jgp/pages/editorial-policies#data-availability-statement.

   Response: We thank the Editor for bringing this requirement to our attention. In accordance with the JGP Data Availability policy, we reviewed the availability of all data underlying the research presented in the manuscript and modified a Data availability.
   The statement specifies that the data underlying Figs. 1–8 and Figs. S1–S4, including individual animal- and cell-level measurements and original experimental recordings, are available from the corresponding author upon request. It also specifies that the uncropped and unprocessed immunoblots underlying Figs. 1, 7, and 8 and Fig. S1 are provided as Figure Source Data files.

Reviewer #1's comments

1. I think it's important when discussing the upstream mechanisms of the altered nNOSβ phosphorylation, to mention mechanisms that may act acutely (mechanical load or require membrane-receptor mediated mechanisms) and those that would be retained chronically when the cardiomycytes are isolated and placed in standard media for a considerable period. The mechanisms that are relevant are ones that are not mediated by an acute mechanisms, that would be retained in vitro. This point needs to be considered in the revised ms.

   Response: We thank the reviewer for this helpful clarification. We agree that our previous discussion did not adequately distinguish acute *in vivo* stimuli from mechanisms capable of accounting for a phosphorylation difference retained after cell isolation. We have revised the Discussion accordingly. We also clarified that physiological experiments were conducted using freshly isolated cardiomyocytes and that samples used for biochemical analyses were pelleted and stored rather than maintained in standard culture medium. Mechanical loading and ligand-dependent adrenergic signaling are now described as possible *in vivo* inputs that may establish a chamber-specific regulatory state, rather than as acute mechanisms operating during the isolated-cell experiments. We further clarify that the difference observed after isolation would require a retained molecular state, such as local kinase-phosphatase regulation, stable microdomain organization, or a persistent post-translational modification.
