# Supplementary material for: Chamber-specific regulation of myofilament Ca2+ sensitivity by nNOSβ via troponin I phosphorylation: Predominance in the right ventricle
Source: J Gen Physiol. 2026 Oct 5;158(6):e202613982. doi: 10.1085/jgp.202613982 (PMC13637471; doi:10.1085/jgp.202613982)
Supplement: Supplementary file 2 [file jgp_202613982_sourcedataf1.pdf]

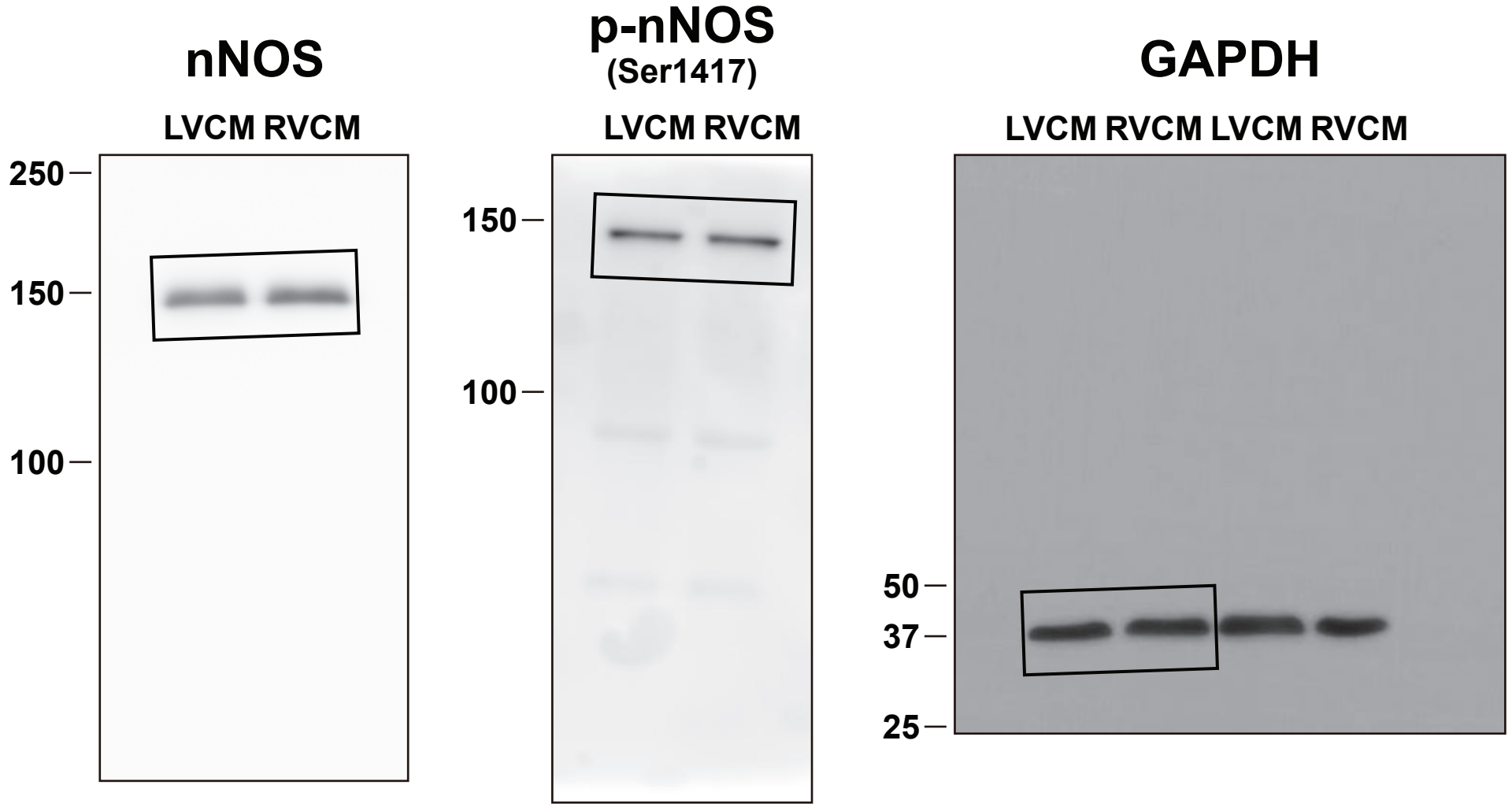
nNOS
LVCM RVCM
250
150
100
p-nNOS
(Ser1417)
LVCM RVCM
150
100
GAPDH
LVCM RVCM LVCM RVCM
50
37
25
