# Supplementary material for: Chamber-specific regulation of myofilament Ca2+ sensitivity by nNOSβ via troponin I phosphorylation: Predominance in the right ventricle
Source: J Gen Physiol. 2026 Oct 5;158(6):e202613982. doi: 10.1085/jgp.202613982 (PMC13637471; doi:10.1085/jgp.202613982)
Supplement: Supplementary file 3 [file jgp_202613982_sourcedataf7.pdf]

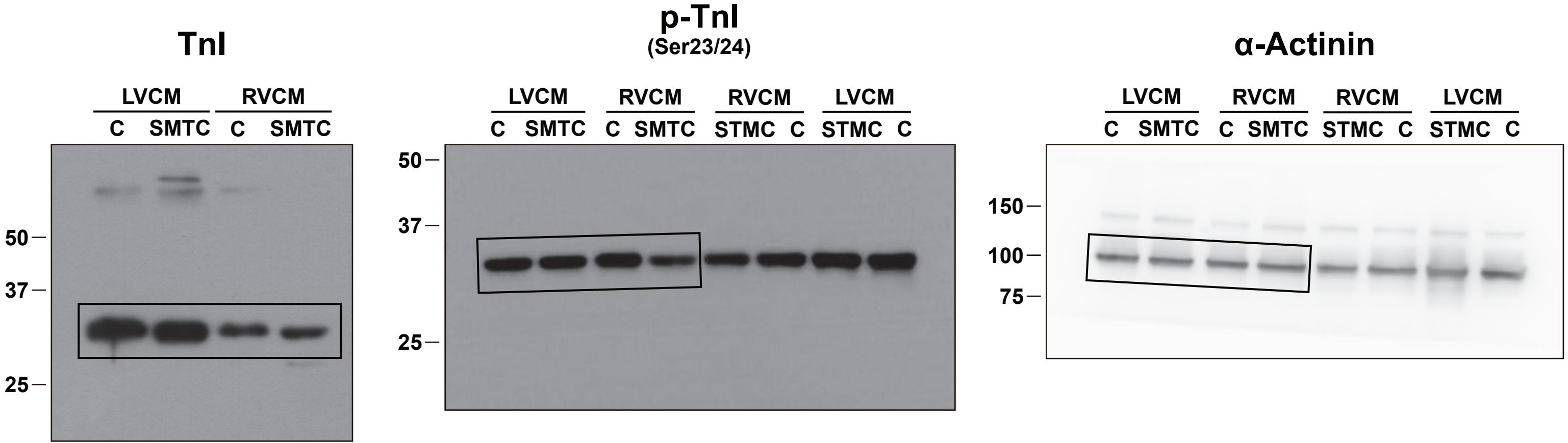
TnI
LVCM
RVCM
C
SMTC
C
SMTC
50
37
25
p-TnI
(Ser23/24)
LVCM
RVCM
RVCM
LVCM
C
SMTC
C
SMTC
STMC
C
STMC
C
50
37
25
α-Actinin
LVCM
RVCM
RVCM
LVCM
C
SMTC
C
SMTC
STMC
C
STMC
C
150
100
75
